# Using Targeted Transcriptome and Machine Learning of Pre- and Post-Transplant Bone Marrow Samples to Predict Acute Graft-versus-Host Disease and Overall Survival after Allogeneic Stem Cell Transplantation

**DOI:** 10.3390/cancers16071357

**Published:** 2024-03-29

**Authors:** Scott D. Rowley, Thomas S. Gunning, Michael Pelliccia, Alexandra Della Pia, Albert Lee, James Behrmann, Ayrton Bangolo, Parul Jandir, Hong Zhang, Sukhdeep Kaur, Hyung C. Suh, Michele Donato, Maher Albitar, Andrew Ip

**Affiliations:** 1 Georgetown University School of Medicine, Washington, DC 20007, USA; 2 John Theurer Cancer Center, Hackensack, NJ 07601, USA; alexandra.dellapia@hmhn.org (A.D.P.); sukhdeep.kaur@hmhn.org (S.K.); michele.donato@hmhn.org (M.D.);; 3 Hackensack Meridian School of Medicine, Nutley, NJ 07110, USA; thomas.gunning@hmhn.org (T.S.G.); michael.pelliccia@hmhn.org (M.P.); albert.lee@hmhn.org (A.L.); james.behrmann@hmhn.org (J.B.); ayrton.bangolo@hmhn.org (A.B.); parul.jandir@hmhn.org (P.J.); 4 Genomic Testing Cooperative, Irvine, CA 92618, USA; hzhang@georgiasouthern.edu (H.Z.); malbitar@genomictestingcooperative.com (M.A.)

**Keywords:** allogeneic transplantation, acute GvHD, post-transplant survival, transcriptomics, machine learning, signaling pathways

## Abstract

**Simple Summary:**

Acute graft-versus-host disease (aGvHD) remains a major cause of morbidity and mortality after allogeneic hematopoietic stem cell transplantation (HSCT), occurring to some degree in over 50% of patients and being a direct cause of death in about 20% of patients. This complication occurs even despite a better understanding of donor selection and GvHD prophylaxis regimens. aGvHD is a complex event in which multiple contributing factors are involved. We performed RNA transcriptome analysis of 1408 genes in bone marrow samples obtained before and after transplantation using machine learning to predict the risk of aGvHD and post-transplant survival for a cohort of patients undergoing HSCT. Differential gene expression identified several signaling pathways in the bone marrow microenvironment that may be major regulators of the complex biology of GvHD, and identified targets of intervention to ameliorate the risk of aGvHD and improve patient survival.

**Abstract:**

Acute graft-versus-host disease (aGvHD) remains a major cause of morbidity and mortality after allogeneic hematopoietic stem cell transplantation (HSCT). We performed RNA analysis of 1408 candidate genes in bone marrow samples obtained from 167 patients undergoing HSCT. RNA expression data were used in a machine learning algorithm to predict the presence or absence of aGvHD using either random forest or extreme gradient boosting algorithms. Patients were randomly divided into training (2/3 of patients) and validation (1/3 of patients) sets. Using post-HSCT RNA data, the machine learning algorithm selected 92 genes for predicting aGvHD that appear to play a role in PI3/AKT, MAPK, and FOXO signaling, as well as microRNA. The algorithm selected 20 genes for predicting survival included genes involved in MAPK and chemokine signaling. Using pre-HSCT RNA data, the machine learning algorithm selected 400 genes and 700 genes predicting aGvHD and overall survival, but candidate signaling pathways could not be specified in this analysis. These data show that NGS analyses of RNA expression using machine learning algorithms may be useful biomarkers of aGvHD and overall survival for patients undergoing HSCT, allowing for the identification of major signaling pathways associated with HSCT outcomes and helping to dissect the complex steps involved in the development of aGvHD. The analysis of pre-HSCT bone marrow samples may lead to pre-HSCT interventions including choice of remission induction regimens and modifications in patient health before HSCT.

## 1. Introduction

Acute graft-versus-host disease (aGvHD) is the leading cause of non-relapse mortality (NRM) in patients after allogeneic hematopoietic stem cell transplantation (allo-HSCT), with 6-month NRM occurring in up to 18% of patients with high-risk aGvHD [1,2]. For several decades, donor selection algorithms and prophylactic post-transplant chemotherapy regimens, with the later addition of calcineurin inhibitors, remained the standard approach to reduce the risk of aGvHD. Despite these routine but imprecise prevention strategies, nearly 40% to 60% of allo-HSCT recipients develop aGvHD, and even patients predicted to be at a low risk of developing severe aGvHD may still develop dire, treatment-refractory, and life-threatening disease [3,4]. 

The pathophysiology of aGvHD involves the activation of donor T-cells, macrophages, monocytes, and neutrophils, and the condition arises when these donated immune cells attack host tissues (e.g., the skin, gastrointestinal tract, and liver), resulting in potentially life-threatening complications [3,5]. These complications range from mild to severe, with the incidence, severity, and morbidity of aGvHD increasing with greater donor-to-host human leukocyte antigen (HLA) disparity. Historical methods for diagnosing aGvHD include symptom evaluation to assign a clinical grade and tissue biopsy to exclude other causes of pathology. However, these strategies are often difficult to quantify, invasive, and lack specificity [6]. The ability to predict the development of aGvHD may allow for a better selection of patients and donors, and the choice of conditioning and immunosuppression regimens. Previous studies in predicting aGvHD focused on the characteristics and effects of donor cells rather than the host microenvironment [7]. Several studies identified that both donor and host characteristics are associated with an increased risk of aGvHD and, more recently, an effort has been made to identify biomarkers associated with or predictive of the development of aGvHD before the onset of clinical manifestations [8,9,10]. For example, the Mount Sinai Acute GvHD International Consortium (MAGIC) algorithm probability (MAP) uses two biomarkers measured pre- and post-transplant to detect changes in the gastrointestinal tract as a response biomarker and correlated these findings with NRM [11]. Still, no method using one or a combination of biomarkers has yet been widely adopted for the prediction or characterization of aGvHD [7,12]. 

Advancements in machine learning are generating interest in developing an integrated, data-driven approach to predict and manage medical events [13,14]. The ability to measure gene expression in various cell populations using transcriptome analysis allows for the exploration of cell pathways involved in various biological events, which is critical as aGvHD is a complex event in which multiple contributing factors are involved. Machine learning employs data-driven statistical modeling approaches that can identify underlying patterns without predefined assumptions. Transcriptome analysis with machine learning has been used to identify gene expression profiles (GEP) including both upregulated and suppressed genes associated with an event. Transcriptome analysis has been used in the analysis of gastrointestinal aGvHD and cutaneous chronic GvHD (cGvHD), identifying potential pathways of T-cell activation that differ from those observed in other inflammatory disease of these organs [15,16]. 

Given the severity of aGvHD as one of the most fatal complications following allo-HSCT, there is a significant need for better and more objective means for the diagnosis and prediction of aGvHD. Using expression data collected by targeted RNA next-generation sequencing (NGS) of bone marrow (BM) samples obtained before and after transplantation, we explored the potential of RNA data using machine learning to predict aGvHD and overall survival (OS).

## 2. Materials and Methods

### 2.1. Patients

This is a retrospective, single-center study of patients undergoing allo-HSCT between 1 November 2019 and 31 August 2022, allowing for at least 6 months of follow-up for the determination of late-onset aGvHD and survival at the time of database closure (29 May 2023). Patients eligible for this study included all patients with either malignant or nonmalignant hematological disease who underwent allo-HSCT and had BM samples analyzed by RNA gene expression using next-generation sequencing (NGS). Institutional Review Board (IRB) approval for this study was obtained from WCG IRB (Pro2020-1406). The study was conducted under the International Conference on Harmonization Good Clinical Practice Guidelines and according to the Declaration of Helsinki. The requirement for patient informed consent (verbal or written) was waived by the IRB as this project represented a non-interventional study using routinely collected data for secondary research purposes. 

Eligibility for transplantation, choice of conditioning and GvHD prophylaxis regimens, and donor and graft sources were not prescribed for enrollment into this study and were per the discretion of the physician caring for the patient. All patients met standard eligibility criteria for allo-HSCT at this center, including age below 80 years, Karnofsky performance status ≥70%, having a readily available HLA matched or mismatched related or unrelated donor (URD), and with appropriate pulmonary, cardiac, hepatic, and renal functions. Stem cell grafts were collected using standard techniques, and no processing of grafts (other than cryopreservation for donor management purposes and red cell depletion of ABO major incompatible bone marrow grafts) was performed. The collection goal for peripheral blood stem cell (PBSC) grafts was >4 × 10^6^ and <10 × 10^6^ CD34+ cells/kg. The target for bone marrow harvesting was >3 × 10^8^ nucleated cells per kilogram, but cell quantities above or below this target were infused without adjustment in number. URD grafts were obtained through the National Marrow Donor Program or similar registries. Per institutional practices, male sex and younger age were given priority in donor selection. Day 0 was defined as the day the cell product infusion was completed.

Patients received pre-transplant conditioning using non-myeloablative (NMA), reduced-intensity (RIC), or myeloablative conditioning (MAC) regimens (Supplemental Table S1) [17]. In general, patients over 60 years of age were conditioned with RIC or NMA regimens. GvHD prophylaxis regimens were determined by the conditioning regimen and donor selected (Supplemental Table S2). Most patients undergoing transplantation using grafts from URD or HLA-matched related donors received a regimen of tacrolimus and methotrexate. A regimen of cyclosporine, sirolimus, and mycophenolate mofetil (MMF) was used for all patients receiving HSC from a non-haploidentical donor after a NMA regimen with single-fraction total body irradiation (TBI, Supplemental Table S1). All patients receiving HSC from related haploidentical donors received a standard regimen of cyclophosphamide, tacrolimus, and MMF (Supplemental Table S2). Two patients treated for aplastic anemia received a GvHD prophylaxis regimen of cyclosporine and methotrexate. Abatacept (Supplemental Table S2) could be added to the GvHD prophylaxis for recipients of haploidentical or unrelated donors. Rabbit anti-thymocyte globulin (ATG, Supplemental Table S2) was added to the regimens for recipients of HLA-matched siblings or unrelated donors receiving busulfan-based MAC regimens and all recipients of reduced-intensity busulfan. 

Patients were hospitalized until neutrophil engraftment, control of any infectious complications, and resolution of severe regimen-related complications. Patients were then seen in post-transplantation follow-up at least weekly through day +100 after transplantation, biweekly through day +180, and then at least monthly through 12 months or until resolution of cGvHD. All patients received a standard antimicrobial prophylaxis regimen starting on initiation of the transplantation conditioning regimen and post-transplant cytokine support (Supplemental Methods S1). Patients were censored from follow-up for aGvHD on day of event for death, relapse, or onset of cGvHD, or infusion of donor lymphocytes (DLI). Patients were censored from follow-up of survival on the day of event for death or relapse or last visit if lost to follow-up.

### 2.2. Diagnosis of aGvHD

Consensus criteria were used to diagnose, stage, and grade aGvHD at least weekly through day +100 after transplantation and then at least every other week through to day +180, with scoring maintained in the electronic medical record [18]. Treatment for aGvHD was not prescribed for this study and was determined by the medical team. Time to aGvHD is the day of onset of any grade; the maximal grade and stage of aGvHD were also recorded for this analysis. No attempt was made to analyze these RNA gene expression data stratified relative to the timing of aGvHD onset and the day of the post-transplant bone marrow sampling. For this analysis, the onset, presence, stage, and grade of aGvHD were adjudicated by a single reviewer (SDR) to confirm these details but using MAGIC criteria [19].

### 2.3. Sample Collection

BM samples for disease staging were usually collected per program standard practice within 28 days before initiation of transplant conditioning and at 84–100 days after allo-HSCT with samples outside these times also included. 

Fresh BM samples were collected in EDTA tubes and transported at room temperature for initial processing. Pre-transplant was defined as BM samples taken from patients before the start of pre-transplant conditioning. Post-transplant was defined as BM samples taken from patients after allo-HSCT. 

### 2.4. RNA Extraction and Next Generation Sequencing (NGS)

BM samples were processed within 72 h of collection. RNA was extracted using an automated Maxwell instrument (Promega, Madison, WI, USA). RNA was first converted to cDNA using random primers. RNA samples were selectively enriched for 1408 cancer-associated genes using the reagents provided in the Illumina^®^ TruSight^®^ RNA pan-cancer panel (Illumina, San Diego, CA, USA; Supplemental Table S3). Sequencing adapters were ligated into the resulting double-stranded cDNA fragments. The coding regions of the expressed genes were captured from this library using sequence-specific probes to create the final library. Sequencing was performed using the Illumina NovaSeq platform (Illumina, San Diego, CA, USA). Ten million reads per sample were performed in a single run, and the read length was 2 × 150 bp. For any sequence to be accepted, it must show >20% reads with splice junction. Expression levels were measured as fragments per kilobase of transcripts per million. 

### 2.5. Machine Learning Algorithm for Predicting aGvHD 

The RNA expression data were used in the machine learning algorithm to predict the presence or absence of aGvHD using either random forest or extreme gradient boosting algorithms. Patients were randomly divided into training (2/3 of patients) and validation (1/3 of patients; Supplemental Table S4a) sets. Randomization was independently performed by computer for the pre- and post-transplant cohorts and, accordingly, different patients from each cohort could be randomly assigned to the training and analysis sets. The Entrez symbols of the genes selected by the algorithm were search for their pathway involvement using the KEGG database and software [20,21,22]. 

### 2.6. Statistical Analysis

Primary clinical endpoints are the development of grade I-IV aGvHD with censoring for relapse or death, and OS censored for relapse. Patients’ characteristics were summarized but not compared between the pre- and post-transplant groups (or between training or analysis subsets). Continuous variables were summarized with median and range and categorical variables were summarized with counts and percentages. 

We developed a machine learning algorithm that first selected the relative genes based on the performance of each gene with cross-validation and based on stability measures using statistical significance tests. The selected genes were then used to predict aGvHD or survival with k-fold cross validation procedures (k = 12). A naïve Bayesian classifier was constructed on the training of k − 1 subsets and tested on the other resting subset. We applied geometric mean naïve Bayesian (GMB) as the classifier for prediction. The details of this machine learning platform were previously reported [23,24].

## 3. Results

### 3.1. Clinical Characteristics

Samples for analysis were available for 167 patients (Table 1), with pre-transplant samples available for 132 patients collected at a median of 29 days before transplantation (range, 14–170 days) and post-transplant samples available for 119 patients collected at a median of 84 days (range, 29–141 days). Patient and donor demographics, transplant diagnoses, conditioning and GvHD regimens, and use of ATG or abatacept are summarized (Table 1, Supplemental Table S4a–c). The median follow-up for the entire group at time of analysis was 344 days (range, 7–925 days). Thirty-six patients suffered disease relapse at a median of 87.5 days (range, 27–718 days) after transplantation. Fifty-nine patients expired of disease relapse or complications of treatment at a median of 165 days (range, 7–560 days). 

### 3.2. Prediction of aGvHD Using Post-Transplant Samples

**Figure 1:**
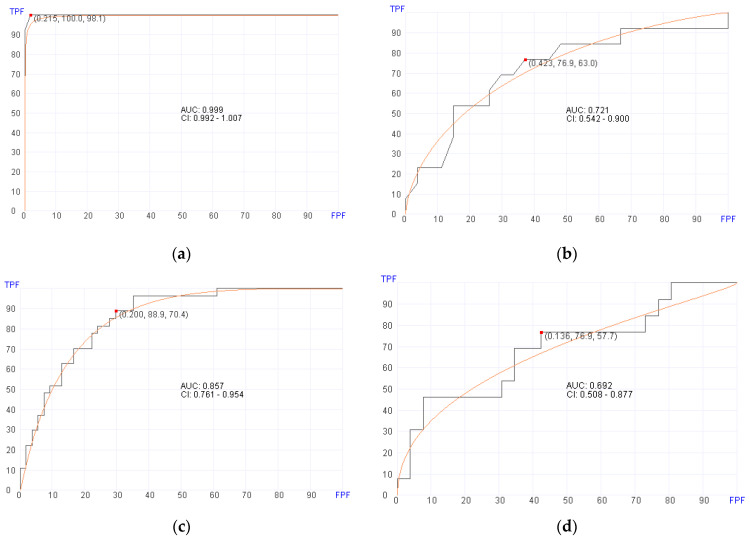
Receiver operator characteristics (ROC) curves for predicting aGvHD in pre- and post-transplant patient cohorts. (**a**) Post-transplant aGvHD prediction using 92 genes (AUC = 0.999, *p* = 0.03) in the training set. (**b**) Post-transplant aGvHD prediction (AUC = 0.721, *p* = 0.03) in the validation set. (**c**) Pre-transplant aGvHD prediction using 400 genes (AUC = 0.857, *p* = 0.02) in the training set. (**d**) Pre-transplant aGvHD prediction using (AUC = 0.692, *p* = 0.02) in the validation set.

Of the 119 patients with post-transplant BM samples available, 80 patients developed aGvHD of any stage (Table 1) at a median of 37.5 days. Using post-transplant BM RNA data, the machine learning algorithm selected 92 genes (Table 2) for predicting aGvHD in the training set with an AUC of 0.999 (95% confidence interval [CI], 0.992–1.007; *p* = 0.03), 100% sensitivity, and 98.1% specificity (Figure 1a). In the validation set, the machine learning algorithm showed an AUC of 0.721 (95% CI, 0.542–0.900; *p* = 0.03), 76.9% sensitivity, and 63% specificity using a cut-off score of 0.423 (Figure 1b). The genes selected by the algorithm for predicting aGvHD appear to play roles involved in PI3K/AKT signaling, MAPK signaling, FOXO signaling, and cancer-associated microRNA.

### 3.3. Prediction of aGvHD Using Pre-Transplant Samples

Of the 132 patients with pre-transplant BM samples available, 87 patients developed aGvHD of any stage (Table 1). Using pre-transplant BM RNA data, the machine learning algorithm selected 400 genes (Supplemental Table S5) for predicting aGvHD in the training set with an AUC of 0.857 (95% CI, 0.761–0.954; *p* = 0.02), 88.9% sensitivity, and 70.4% specificity (Figure 1c). In the validation set, the machine learning algorithm showed an AUC of 0.692 (95% CI, 0.508–0.877; *p* = 0.02), 76.9% sensitivity, and 57.7% specificity using a cutoff of 0.136 (Figure 1d).

### 3.4. Prediction of Overall Survival (OS) Using Post-Transplant Samples

**Figure 2:**
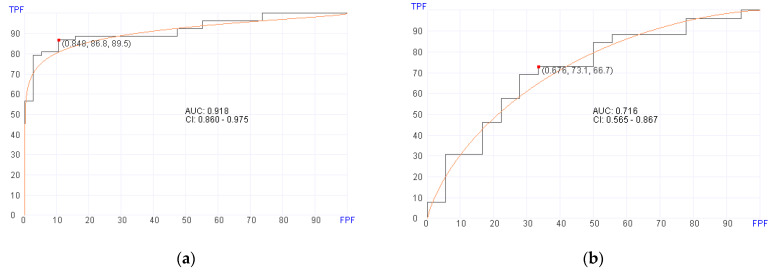

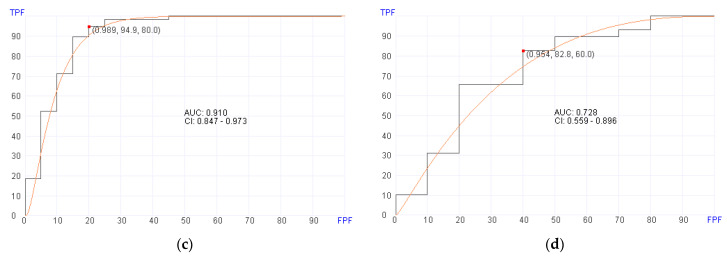
Receiver operator characteristics (ROC) curves for predicting overall survival in pre- and post-transplant patient cohorts. (**a**) Post-transplant survival prediction using 20 genes (AUC = 0.910, *p* = 0.02) in the training set. (**b**) Post-transplant survival prediction using 20 genes (AUC = 0.716, *p* = 0.02) in the validation set. (**c**) Pre-transplant survival prediction using 700 genes (AUC = 0.910, *p* = 0.02) in the training set. (**d**) Pre-transplant survival prediction using 700 genes (AUC = 0.728, *p* = 0.02) in the validation set.

Of the 119 patients with post-transplant BM samples available, 87 were alive at a median follow up of 14 months at the time of data analysis. Using post-transplant BM RNA data, the machine learning algorithm selected 20 genes (Table 3) for predicting OS in the training set with an AUC of 0.918 (95% CI, 0.860–0.975; *p* = 0.02), 86.8% sensitivity, and 89.5% specificity (Figure 2a). In the validation set, the machine learning algorithm showed an AUC of 0.716 (95% CI, 0.565–0.867; *p* = 0.02), 73.1% sensitivity, and 66.7% specificity using a cut-off score at 0.676 (Figure 2b). The genes that are selected by the algorithm for predicting survival included genes involved in MAPK signaling pathway and chemokine signaling.

### 3.5. Prediction of Overall Survival (OS) Using Pre-Transplant Samples

Using pre-transplant BM RNA data, the machine learning algorithm selected 700 genes (Supplemental Table S6) for predicting aGvHD in the training set with an AUC of 0.910 (95% CI, 0.847–0.973; *p* = 0.02), 94.9% sensitivity, and 80% specificity (Figure 2c). In the validation set, the machine learning algorithm showed an AUC of 0.728 (95% CI, 0.559–0.896; *p* = 0.02), 82.8% sensitivity, and 60% specificity using a cut-off score at 0.954 (Figure 2d).

## 4. Discussion

In this study, we analyzed gene expression profiles of BM samples that were obtained before and after transplantation for patients undergoing allo-HSCT. Patients were randomly assigned into training and validation cohorts independently for the pre- and post-HSCT samples. Using pre- and post-transplant BM samples, we developed machine learning algorithms that selected for a number of genes to predict aGvHD and OS. 

The expressions of more than 1400 genes were used in this study to evaluate bone marrow environment in pre- and post-transplant samples. Using a machine learning algorithm is necessary to adjust for the multiple variables that may contribute to the prediction of the presence or absence of aGvHD and eliminate statistically insignificant markers. A Bayesian approach is specifically appropriate to use when the number of cases is limited. Furthermore, a Bayesian approach allows us to define the specific biomarkers that are relevant for the classification so the classification is less of a “blackbox” as compared with other classifiers such as random forest or extreme gradient booster. The expression of only 20 genes were adequate for the prediction of survival using post-transplant samples, reflecting significant changes in bone marrow that are detrimental to the survival of the patients. Similarly, for predicting aGvHD in post-transplant bone marrow samples, only 92 genes are needed. In contrast, for predicting aGvHD and survival in pre-transplant bone marrow samples, 400 genes and 700 genes are needed, respectively. This suggests that pre-transplant, there are more bone marrow microenvironment factors that play a role in future development of aGvHD and subsequent survival. 

The post-HSCT samples showed a high correlation with the presence of aGvHD. These samples were mostly obtained after the onset of aGvHD and, therefore, we cannot propose that this analysis serves as a biomarker predictive for the development of this complication of HSCT. Yet identification of the involved pathways may facilitate the development of GvHD prophylaxis regimens beyond the currently widely used calcineurin-based prophylaxis regimens that could be effective in suppressing the onset of aGvHD, facilitating the development of tolerance, and reducing the risk of off-target toxicities. This analysis may be equally valuable in dissecting the pathways involved with aGvHD, leading to more-specific non-steroid treatments including targeted treatments to manage steroid-resistant aGvHD (or cGvHD). Examples of such targeted therapies includes inhibition of the Janus kinase (JAK) [25,26,27], Bruton’s tyrosine kinase (BTK) [28,29,30], and Rho-associated coiled-coil-containing protein kinase 2 (ROCK2) [31] signaling pathways, each of which now have FDA-approved medications for management of steroid-refractory acute and/or chronic GvHD [32]. Our findings are in agreement with other reports such as the association of microRNA with acute and chronic GvHD [33].

The results of the pre-HSCT sample analysis also show that transcriptome analysis of the bone marrow microenvironment is predictive of aGvHD and OS, although we could not define specific signaling pathways. With further investigation, it may be possible to use transcriptome analysis to develop biomarker(s) predictive of the development of aGvHD and OS, allowing for modifications in the planned transplant treatment plan, and thereby improving transplant outcomes [4]. We hypothesize that transcriptome analysis early in the course of the disease could lead to modifications in patient care during initial remission induction and consolidation cycles before referral for transplantation. We further hypothesize that our findings may correspond to the immune microenvironment possibly being influenced by microbiome effects on transplant outcomes, for example, with the potential for prevention of aGvHD [34,35]. The pre-transplant BM specimens would be affected by therapies given in the control of disease in anticipation of allo-HSCT.

Our analysis of OS using post-transplant samples is complicated by the presence of aGvHD in a large proportion of the patient cohort, and we cannot ascertain, using this dataset, if the signaling pathways associated with OS are distinct from or overlap the signaling pathways associated with aGvHD. We also did not attempt to correlate our analysis with the onset or severity of cGvHD. That transcriptome analysis of the pre-transplant samples also predicts OS demands further, in-depth analysis of the patient populations being referred for transplantation, hopefully leading to improvements in the transplant process.

Numerous investigators are exploring the gene expression profile associated with the immunological GvHD and graft-versus-disease (GvL) events of HSCT. Such studies led to clinical studies of JAK, BTK, and ROCK2 inhibitors, resulting in FDA approval for these therapies [22,23,24,25,26,27,28,29]. Most of the studies, however, combine analysis of gene expression profile of certain lymphocyte populations to explore the biology of immunological reconstitution after HSCT, such as the study by McCurdy et al. of patients receiving PTCy for GvHD prophylaxis using machine learning and RNASeq analysis of blood lymphocyte subsets at day +28 after transplantation, which found 56 differentially expressed genes (DEGs) in regulatory T cells in patients who developed aGvHD [36]. Our analysis, especially of pre-transplant samples, may identify signaling pathways that will lead to more in-depth analysis of the immunological events occurring during HSCT.

The primary limitation of our study is the single-center, retrospective design and the relatively small study populations in both the training and validation cohorts. The primary advantage in this study is the large variation in patient and treatment characteristics, showing that this approach may be valid over a wide range of patients. The strong correlation across a number of patient variables including diagnosis, donor type, transplant conditioning regimen, and GvHD prophylaxis emphasizes the strength of our findings. Furthermore, our analysis of over 1400 genes is not restricted to specific signaling pathways that have been the subject of study in the previously reported analyses of acute and chronic GvHD. While we do not propose that eligibility for transplant should be based at this time on testing bone marrow samples using the approach used in this study, the current study points out the importance of the pre-transplant bone marrow microenvironment in the potential of developing aGvHD and overall survival. Further studies are necessary and appropriate to explore the bone marrow microenvironment and to improve the management of aGvHD.

## 5. Conclusions

In conclusion, our study shows that targeted transcriptome analysis of pre- and post-transplant BM samples can predict aGvHD and OS with relatively high accuracy when a large number of genes are used. Although the accuracy of this prediction is higher when post-transplant transcriptomic data are used, the pre-transplant BM microenvironment is very important and relevant for the future development of aGvHD and for overall survival. This confirms that both the host BM microenvironment and the donor cells may play a significant role in the development of aGvHD and OS in patients undergoing allo-HSCT. Although preliminary, our study demonstrates expression data collected by targeted RNA NGS using machine learning can predict aGvHD and survival. Future studies are needed to validate our findings.

## Figures and Tables

**Table 1:** Patient characteristics.

Characteristics	All Patients N = 167 *n* (%)	Pre-Transplant Patients N = 132 *n* (%)	Post-Transplant Patients N = 119 *n* (%)
Recipient age, median years (range)	63.0 (20.8–79.0)	64.0 (20.8–79.0)	63.7 (20.8–79.0)
Recipient sex			
Male	89 (53)	71 (54)	62 (52)
Indication for allo-HSCT			
ALL	20 (12)	15 (11)	15 (13)
AML	57 (34)	40 (30)	47 (39)
CML	3 (2)	2 (2)	3 (3)
MDS	44 (26)	37 (28)	31 (26)
Myelofibrosis/CMML	32 (19)	27 (20)	19 (16)
SAA	4 (2)	4 (3)	1 (1)
NHL	7 (4)	7 (5)	3 (3)
Graft source			
Bone marrow	130 (78)	30 (23)	23 (19)
Peripheral blood	37 (22)	102 (77)	96 (81)
HLA compatibility			
Unrelated donor HLA match	84 (50)	64 (48)	65 (55)
Unrelated donor HLA mismatch	17 (10)	13 (10)	13 (11)
HLA matched related donor	14 (8)	11 (8)	9 (8)
Related donor, haploidentical	52 (31)	44 (33)	32 (27)
Donor age, median years (range)	28.3 (14.0–63.7)	28.1 (17.6–63.7)	28.0 (14.0–59.0)
Donor age < 35 years	120 (72)	98 (74)	89 (75)
Donor sex			
Male	109 (65)	82 (62)	77 (65)
Conditioning regimen			
Myeloablative	43 (26)	30 (23)	33 (28)
Non-myeloablative	47 (28)	41 (31)	31 (26)
Reduced intensity	77 (46)	61 (46)	55 (46)
aGvHD prophylaxis regimen			
PtCy	73 (44)	62 (47)	49 (41)
TacMTX	75 (45)	55 (42)	58 (49)
RapaCspMMF	19 (11)	15 (11)	12 (10)
Addition of abatacept	21 (13)	18 (14)	16 (13)
Addition of anti-thymocyte globulin	38 (23)	26 (20)	31 (26)
Diagnosed with aGvHD			
Stage 1–4	109 (65)	87 (66)	80 (67)
Stage 3–4	7 (4)	6 (5)	1 (1)
Site of aGvHD			
Gastrointestinal	65 (39)	51 (71)	49 (40)
Lower	17 (10)	12 (30)	10 (8)
Upper	48 (29)	39 (41)	39 (32)
Liver	5 (3)	4 (3)	4 (3)
Skin	63 (38)	54 (41)	51 (43)

Allo—allogeneic; ALL—acute lymphoblastic leukemia; AML—acute myeloid leukemia; CML—chronic myeloid leukemia; CMML—chronic myelomonocytic leukemia; CSPMMF—cyclosporine and mycophenolate mofetil, MDS—myelodysplastic syndromes; NHL—non-Hodgkin’s lymphoma; PtCy—post-transplant cyclophosphamide; RapaCSPMMF—rapamycin, cyclosporine, and mycophenolate mofetil; RIC—reduced intensity conditioning; SAA—severe aplastic anemia; TacMTX—tacrolimus and methotrexate.

**Table 2:** 92 Genes Predicting aGvHD in Post-transplant Samples.

92 Genes Predicting GvHD			
1–23	24–46	47–69	70–92
DUSP2	CDKN1A	NEURL1	SUZ12
CD22	TFRC (CD71)	TNFRSF17 (BCMA)	TRIM33
FLNA	DLL3	BCL7A	CDK9
PAX8	SSBP2	YTHDF2	FLYWCH1
ARHGEF12	TRAF3	KIF5B	HIST1H2BC
AKAP9	PSIP1	IRS1	MAPK1
DLL4	43717SEPT9	DGKZ	RAC2
AIP	SPTBN1	CENPU	TCF7L2
CDC14B	HIST1H2AC	STIL	USP42
FOXO3	TFDP1	XKR3	FGFR1OP
EGR4	TRAF5	CCT6B	MTCP1
MUTYH	BACH2	CD28	PTPRO
SS18L1	TNFRSF10D	OLIG1	SH3D19
PRKCG	SLC45A3	CCND2	CTDSP2
HOOK3	NACA	GID4	ID3
TCEA1	ASPH	STYK1	SMAP1
UBE2C	ZBTB16	ATF3	STL
FIGF	EPHA2	FGF9	TAL1
TOP1	APOD	ZNF703	DNMT3A
DTX1	KAT2B	AKAP12	IKBKE
TNF	ETV5	PTCRA	IKZF3
CCNE1	FGF13	SMAD6	AKT3
BAIAP2L1	FLT3LG	DNAJB1	HSPA4

Shown are the 92 genes identified in post-transplant marrow samples that associated with the development of aGvHD. Genes are listed in order of expression.

**Table 3:** 20 Genes Predicting Survival in Post-transplant Samples.

Genes Predicting Survival	
1–10	11–20
ATIC	TGFBI
PLAG1	BRSK1
CD36	KIT (CD117)
HSP90AB1	MSH6
DNMT1	HIST1H1D
WDR1	HEY1
CDC14A	FOXO1
MALT1	PRKCA
SP3	CCNB1IP1
MAP3K14	FANCC

Shown are the 20 genes identified in post-transplant marrow samples that associated with the overall survival. Genes are listed in order of expression.

## Data Availability

The complete datasets used and/or analyzed during this study are available from the corresponding author upon request. Requests can be made through the corresponding author or directly to representatives of Hackensack Meridian Health (Scott D. Rowley; Email: sdr62@georgetown.edu).

## Supplementary Materials

The following supporting information can be downloaded at: https://www.mdpi.com/article/10.3390/cancers16071357/s1, Method S1: Post-transplant Supportive Care Policies and Procedures; Table S1: Description of Conditioning Regimens; Table S2: GvHD Prophylaxis Regimens; Table S3: List of Genes Included in the Analysis of Bone Marrow Samples; Table S4a: Patient and Donor Demographics; Table S4b: Transplant Source and Conditioning and GvHD Regimens; Table S4c: aGvHD and Survival; Table S5: 400 Genes Predicting aGvHD in Pre-transplant Samples; Table S6: 700 Genes Predicting Overall Survival in Pre-transplant Samples
