# Supplementary material for: Using Targeted Transcriptome and Machine Learning of Pre- and Post-Transplant Bone Marrow Samples to Predict Acute Graft-versus-Host Disease and Overall Survival after Allogeneic Stem Cell Transplantation
Source: Cancers (Basel). 2024 Mar 29;16(7):1357. doi: 10.3390/cancers16071357 (PMC11011125; doi:10.3390/cancers16071357)
Supplement: Supplementary file 1 [file cancers-16-01357-s001.zip › cancers-2905585-supplementary.pdf]

Supplementary Material

Methods S1: ***Post-transplant Supportive Care Policies and Procedures***

Patients were hospitalized until neutrophil engraftment, control of any infectious complications, and resolution of severe regimen-related complications. After hospital discharge, patients were followed at least weekly through day +100, biweekly through day +180, and then at least monthly through 12 months after transplantation. All patients received starting on initiation of the transplantation conditioning regimen a standard antimicrobial prophylaxis regimen consisting of valacyclovir 500 mg po twice daily continued for at least 12 months, ciprofloxacin 500 mg po twice daily until neutrophil engraftment, and fluconazole 400 mg po daily through day 84 or discontinuation of corticosteroid therapy, if given. Posaconazole or voriconazole was substituted for fluconazole for patients with GvHD requiring higher doses or prolonged courses of corticosteroids. Letermovir, 480 mg daily for cytomegalovirus (CMV) prophylaxis commencing at transplant admission through at least day +100 (or longer per physician discretion) was administered to CMV seropositive patients. Pneumocystis pneumonia prophylaxis using trimethoprim/sulfamethoxazole, atovaquone, or dapsone was initiated after engraftment achieved and continued until completion of any GvHD prophylaxis and/or treatment. All patients received G-CSF 5 mcg/kg/day subcutaneously rounded to vial size starting on day +5 until achieving a sustained absolute neutrophil count (ANC) of $\geq 0.5 \times 10^9$/L. Leukocyte-depleted and irradiated blood products were given prophylactically for hemoglobin <7 gm/dL or platelet count $<10 \times 10^9$/L.

Table S1: *Description of Conditioning Regimens* 1

Conditioning Regimen Intensity	Regimen	Donor
Myeloablative (MAC)		
• FluBu	Busulfan 130 mg/m^2 IV daily x4 on days -4 to -2; fludarabine 30 mg/m^2 IV daily x4 on days -4 to -2.	Unrelated, HLA-matched related
• FluTBI	Fludarabine 30 mg/m^2 IV daily x4 on days -4 to -2; TBI 150 cGy bid on days -4 to -1 (12 Gy total dose).	Related haploidentical
• TBICy	TBI 150 cGy bid on days -7 to -4 (12 Gy total dose); cyclophosphamide 60 mg/kg daily x2 on days -3, -2.	Unrelated, HLA-matched related
Reduced Intensity RIC		
• FluBu2	Fludarabine 30 mg/m^2 IV daily x5 on days -7 to -3; busulfan 130 mg/m^2 IV daily x2 on days -4 to -3; rATG 2 mg/kg iv daily x3 on days -4 to -2.	Unrelated, HLA-matched related
• FluMel	Fludarabine 30 mg/m^2 iv daily x4 on days -5 to -2; melphalan 140 mg/m^2 iv x1 on day -2.	Unrelated, HLA-matched related
• FluMelTBI	Melphalan 100 mg/m^2 on day -6; fludarabine 40 mg/m^2 iv daily x4 on days -5 to -2; TBI, 2 Gy x1 fraction on day -1.	Related haploidentical
Non-myeloablative (NMA)		
• FluTBI (300)	Fludarabine 30 mg/m^2 iv daily x3 on days -4, -3, and -2; TBI 3 Gy x1 fraction on day 0	Unrelated, HLA-matched related
• FluCyTBI	Fludarabine 30 mg/m^2 i.v. daily x5 on days -6 to -2; cyclophosphamide 14.5 mg/kg i.v. daily x2 on days -6 to -5; TBI 2 Gy x1 fraction on day -1.	Related haploidentical
• CyeATG	Cyclophosphamide 50 mg/kg i.v. daily x4 on days -5 to -2; equine ATG 30 mg/kg i.v. daily x3 on days -4 to -2.	Related matched donor, aplastic anemia

2

Table S2: *GvHD Prophylaxis Regimens*

GvHD Prophylaxis		Indications
• TacMtx	Methotrexate 5 mg/m^2 IV on days +1, 3, 6, 11; tacrolimus 1 mg i.v. daily starting day -2, adjusted to achieve a therapeutic level of 5–15 ng/mL, tapered off by day 180	Unrelated donors: MA regimens<br><br>Related HLA matched donors: MA and RIC regimens
• PTCy	Cyclophosphamide 50 mg/kg iv days +3, +4; mycophenolate mofetil 15 mg/kg bid days +5 to +35; tacrolimus 1 mg i.v.. daily, adjusted to achieve a therapeutic level of 5–15 ng/mL, tapered off by day 180.	Related haploidentical donors, all regimens
• RapaCSPMMF	Sirolimus 2 mg once daily on day −3 and adjusted to maintain trough levels between 3 and 12 ng/mL through day 180, followed by taper through day 365; cyclosporine 5 mg/kg orally twice daily on day −3 and continued to day 150 and then tapered off by day 180; mycophenolate mofetil 15 mg/kg three times daily from day 0 to 30, then twice daily to day 100, and tapered to day 150	Unrelated donors: Nonmyeloablative regimen<br><br>Related HLA matched donors: Nonmyeloablative regimen
• Abatacept	10 mg/m^2 IV days -1, +5, +14, +28	Unrelated donors, TacMTX prophylaxis<br><br>Haploidentical donors, PTCy prophylaxis
• rATG	4 mg/kg IV total divided daily doses days -3 to -1<br><br>2 mg/kg IV daily x3 days -4 to -2	Busulfan myeloablative regimen. Melphalan non-myeloablative regimen with TacMTX<br><br>Busulfan reduced intensity regimen

Genes Included in Analysis					
**1-250**	**251-500**	**501-750**	**751-1000**	**1001-1250**	**1251-1408**
ABCC3	CNBP	GAS1	MAP2	PPP2R1A	TCF12
ABI1	CNOT2	GAS5	MAP2K1	PPP2R1B	TCF3
ABL1	CNTN1	GAS7	MAP2K2	PPP2R2B	TCF7L2
ABL2	CNTRL	GATA1	MAP2K3	PPP2R4	TCL1A
ABLIM1	COG5	GATA2	MAP2K4	PPP3CA	TCL6
ACACA	COL11A1	GATA3	MAP2K5	PPP3CB	TCTA
ACE	COL1A1	GATA6	MAP2K6	PPP3CC	TEAD1
ACER1	COL1A2	GBP2	MAP2K7	PPP3R1	TEAD2
ACKR3	COL3A1	GDF6	MAP3K1	PPP3R2	TEAD3
ACSBG1	COL6A3	GFAP	MAP3K14	PPP4C	TEAD4
ACSL3	COL9A3	GHR	MAP3K6	PQLC3	TEC
ACSL6	COMMD1	GID4	MAP3K7	PRCC	TENM1
ACVR1B	COX6C	GIT2	MAPK1	PRDM1	TERF1
ACVR1C	CPNE1	GLI1	MAPK3	PRDM16	TERF2
ACVR2A	CPS1	GLI3	MAPK8	PRDM7	TERT
ADD3	CPSF6	GMPS	MAPK8IP2	PRF1	TET1
ADM	CRADD	GNA11	MAPK9	PRG2	TET2
AFF1	CREB1	GNA12	MAPRE1	PRICKLE1	TFAP2A
AFF3	CREB3L1	GNA13	MATK	PRKACA	TFDP1
AFF4	CREB3L2	GNAI1	MAX	PRKACG	TFE3
AGR3	CREBBP	GNAQ	MB21D2	PRKAR1A	TFEB
AHCYL1	CRKL	GNAS	MBNL1	PRKCA	TFG
AHI1	CRLF2	GNG4	MBTD1	PRKCB	TFPT
AHR	CRTC1	GOLGA5	MCL1	PRKCD	TFRC
AHRR	CRTC3	GOPC	MDC1	PRKCG	TGFB2
AIP	CSF1	GOSR1	MDH1	PRKDC	TGFB3
AK2	CSF1R	GOT1	MDM2	PRKG2	TGFBI
AK5	CSF3	GPC3	MDM4	PRMT1	TGFBR2
AKAP12	CSF3R	GPHN	MDS2	PRMT8	TGFBR3
AKAP6	CSNK1G2	GPR124	MEAF6	PROM1	THADA
AKAP9	CSNK2A1	GPR128	MECOM	PRRX1	THBS1
AKR1C3	CTCF	GPR34	MED12	PRRX2	THRAP3
AKT1	CTDSP2	GRB10	MEF2B	PRSS8	TIAM1
AKT2	CTLA4	GRB2	MEF2BNB-MEF2B	PSD3	TIRAP
AKT3	CTNNA1	GRHPR	MEF2C	PSEN1	TLL2
ALDH1A1	CTNNB1	GRID1	MEF2D	PSIP1	TLR4
ALDH2	CTNND2	GRIN2A	MELK	PSMD2	TLX1
ALDOC	CTRB1	GRIN2B	MEN1	PTBP1	TLX3
ALK	CTRB2	GRM1	MET	PTCH1	TMEM127
AMER1	CTSA	GRM3	METTL18	PTCRA	TMEM230
AMH	CUX1	GSK3B	METTL7B	PTEN	TMEM30A
ANGPT1	CXCL8	GSN	MFNG	PTGS2	TMPRSS2

ANKRD28	CXCR4	GSTT1	MGEA5	PTK2	TNC
ANLN	CXXC4	GTF2I	MGMT	PTK2B	TNF
APC	CYFIP2	GTSE1	MIB1	PTK7	TNFAIP3
APH1A	CYLD	H2AFX	MIPOL1	PTPN11	TNFRSF10B
APLP2	CYP1B1	H3F3A	MIR1260B	PTPN2	TNFRSF10D
APOD	CYP2C19	HAS2	MIR4321	PTPN6	TNFRSF11A
AR	DAB2IP	HDAC1	MIR4683	PTPRA	TNFRSF14
ARAF	DACH1	HDAC2	MIR4758	PTPRK	TNFRSF17
ARFRP1	DACH2	HDAC3	MIR6515	PTPRO	TNFRSF6B
ARHGAP20	DAXX	HDAC4	MIR6752	PTPRR	TOP1
ARHGAP26	DCLK2	HDAC5	MIR6765	PTTG1	TOP2A
ARHGEF12	DCN	HDAC6	MIR6795	PVT1	TOP2B
ARHGEF7	DDB2	HDAC7	MIR6857	RABEP1	TP53
ARID1A	DDIT3	HECW1	MIR6894	RAC1	TP53BP1
ARID2	DDR2	HEPH	MITF	RAC2	TP63
ARIH2	DDX10	HERPUD1	MKI67	RAC3	TP73
ARNT	DDX20	HES1	MKL1	RAD21	TPD52L2
ARRDC4	DDX39B	HES5	MKL2	RAD50	TPM3
ASMTL	DDX3X	HEY1	MLF1	RAD51	TPM4
ASPH	DDX5	HGF	MLH1	RAD51B	TPO
ASPSCR1	DDX6	HHEX	MLLT1	RAD51C	TPR
ASTN2	DEK	HIF1A	MLLT10	RAD51D	TRAF2
ASXL1	DGKB	HIP1	MLLT11	RAD52	TRAF3
ATF1	DGKI	HIPK1	MLLT3	RAF1	TRAF5
ATF3	DGKZ	HIPK2	MLLT4	RALGDS	TRHDE
ATG13	DICER1	HIST1H1C	MLLT6	RANBP17	TRIM24
ATG5	DIRAS3	HIST1H1D	MMP7	RANBP2	TRIM27
ATIC	DIS3L2	HIST1H1E	MMP9	RAP1GDS1	TRIM33
ATL1	DKK1	HIST1H2AC	MN1	RARA	TRIP11
ATM	DKK2	HIST1H2AG	MNAT1	RASAL1	TRPS1
ATP1B4	DKK4	HIST1H2AL	MNX1	RASGEF1A	TSC1
ATP6V1G2-DDX39B	DLEC1	HIST1H2AM	MPL	RASGRF1	TSC2
ATP8A2	DLL1	HIST1H2BC	MRE11A	RASGRF2	TSHR
ATR	DLL3	HIST1H2BJ	MSH2	RASGRP1	TTK
ATRNL1	DLL4	HIST1H2BK	MSH3	RB1	TTL
ATRX	DMRT1	HIST1H2BO	MSH6	RBM15	TUSC3
AURKA	DMRTA2	HIST1H3B	MSI2	RBM6	TYK2
AURKB	DNAJB1	HIST1H4I	MSN	RCHY1	TYMS
AUTS2	DNM1	HLF	MTCP1	RCOR1	U2AF1
AXIN1	DNM2	HMGA1	MTOR	RCSD1	U2AF2
AXL	DNM3	HMGA2	MTUS2	RECQL4	UBE2B
BACH1	DNMT1	HMGB1	MUC1	REEP3	UBE2C
BACH2	DNMT3A	HMGN2P46	MUTYH	RELA	UFC1
BAG4	DOCK1	HNF1A	MYB	RELN	UFM1
BAIAP2L1	DOT1L	HNRNPA2B1	MYBL1	RERG	USP16
BAP1	DPM1	HOOK3	MYC	RET	USP42

BARD1	DPYD	HOXA10	MYCL	RGS7	USP5
BAX	DST	HOXA11	MYCN	RHBDF2	USP6
BAZ2A	DTX1	HOXA13	MYD88	RHOA	USP7
BCAS3	DTX4	HOXA3	MYH11	RHOD	VCAM1
BCAS4	DUSP2	HOXA9	MYH9	RHOH	VEGFA
BCL10	DUSP22	HOXC11	MYO18A	RICTOR	VEGFC
BCL11A	DUSP26	HOXC13	MYO1F	RLTPR	VGLL3
BCL11B	DUSP9	HOXD11	NAB2	RMI2	VHL
BCL2	DUX2	HOXD13	NACA	RNF213	VTI1A
BCL2A1	DUX4	HOXD9	NAPA	RNF43	WASF2
BCL2L1	DUX4L2	HRAS	NAV3	ROBO1	WDFY3
BCL2L2	DUX4L4	HSP90AA1	NBEAP1	ROBO2	WDR1
BCL3	E2F1	HSP90AB1	NBN	ROS1	WDR18
BCL6	EBF1	HSPA1A	NBR1	RPA3	WDR70
BCL7A	ECT2L	HSPA1B	NCAM1	RPL22	WDR90
BCL9	EDIL3	HSPA2	NCKIPSD	RPN1	WEE1
BCOR	EDNRB	HSPA4	NCOA1	RPN2	WHSC1
BCORL1	EED	HSPA5	NCOA2	RPS21	WHSC1L1
BCR	EEFSEC	HTRA1	NCOA3	RPS6KA1	WIF1
BDNF	EGF	HUWE1	NCOA4	RPS6KA2	WISP3
BHLHE22	EGFR	IBSP	NCOR2	RPS6KA3	WNT10A
BICC1	EGR1	ICAM1	NCSTN	RPTOR	WNT10B
BIN1	EGR2	ICK	NDC80	RREB1	WNT11
BIRC3	EGR3	ID1	NDE1	RRM1	WNT16
BIRC6	EGR4	ID3	NDRG1	RRM2B	WNT2B
BIVM-ERCC5	EIF4A2	ID4	NDUFAF1	RTEL1	WNT3
BLM	EIF4E	IDH1	NEDD4	RTEL1-TNFRSF6B	WNT4
BMP4	ELF4	IDH2	NEURL1	RTN3	WNT5B
BMPR1A	ELK4	IFNG	NF1	RUNX1	WNT6
BRAF	ELL	IFRD1	NF2	RUNX1T1	WNT7B
BRCA1	ELN	IGF1	NFATC1	RUNX2	WNT8B
BRCA2	ELOVL2	IGF1R	NFATC2	RYR3	WRN
BRD1	ELP2	IGFBP2	NFE2L2	S1PR2	WSB1
BRD3	EML1	IGFBP3	NFIB	SARNP	WT1
BRD4	EML4	IKBKB	NFKB1	SBDS	WWOX
BRIP1	ENPP2	IKBKE	NFKB2	SCN8A	WWTR1
BRSK1	EP300	IKZF1	NFKBIA	SDC4	XBP1
BRWD3	EP400	IKZF2	NGF	SDHA	XIAP
BTBD18	EPC1	IKZF3	NGFR	SDHAF2	XKR3
BTG1	EPCAM	IL12RB2	NIN	SDHB	XPA
BTG2	EPHA10	IL13	NIPBL	SDHC	XPC
BTK	EPHA2	IL13RA2	NKX2-1	SDHD	XPO1
BTLA	EPHA3	IL15	NKX2-5	SEC31A	XRCC6
BUB1B	EPHA5	IL1B	NOD1	2-Sep	YAP1
C10orf55	EPHA7	IL1R1	NODAL	5-Sep	YPEL5
C11orf1	EPHB1	IL1RAP	NONO	6-Sep	YTHDF2

C11orf30	EPHB6	IL2	NOS3	9-Sep	YWHAE
C11orf54	EPO	IL21R	NOTCH1	SERP2	YY1AP1
C11orf95	EPOR	IL2RA	NOTCH2	SERPINE1	ZBTB16
C2CD2L	EPS15	IL3	NOTCH3	SERPINF1	ZC3H7A
C2orf44	ERBB2	IL6	NOTCH4	SET	ZC3H7B
C3orf27	ERBB3	IL7R	NPM1	SETBP1	ZFP64
CACNA1F	ERBB4	INHBA	NPM2	SETD2	ZFPM2
CACNA1G	ERC1	INPP4A	NR3C1	SETD7	ZFYVE19
CACNA2D3	ERCC1	INPP4B	NR4A3	SF3B1	ZIC2
CAD	ERCC2	INPP5A	NR6A1	SFPQ	ZMIZ1
CALR	ERCC3	INPP5D	NRAS	SFRP2	ZMYM2
CAMK2A	ERCC4	IQCG	NSD1	SFRP4	ZMYM3
CAMK2B	ERCC5	IRF1	NT5C2	SGK1	ZMYND11
CAMK2G	ERCC6	IRF2BP2	NTF3	SGPP2	ZNF207
CAMTA1	ERG	IRF4	NTF4	SH2D5	ZNF217
CANT1	ERLIN2	IRF8	NTRK1	SH3BP1	ZNF24
CAPRIN1	ESR1	IRS1	NTRK2	SH3D19	ZNF331
CAPZB	ETS1	IRS2	NTRK3	SH3GL1	ZNF384
CARD11	ETS2	IRS4	NUMA1	SH3GL2	ZNF444
CARM1	ETV1	ITGA5	NUP107	SHC1	ZNF521
CARS	ETV4	ITGA7	NUP214	SHC2	ZNF585B
CASC5	ETV5	ITGA8	NUP93	SIK3	ZNF687
CASP3	ETV6	ITGAV	NUP98	SIN3A	ZNF703
CASP7	EWSR1	ITGB3	NUTM1	SIRT1	ZRSR2
CASP8	EXOSC6	ITK	NUTM2A	SKP2	
CAV1	EXT1	ITPKA	NUTM2B	SLC1A2	
CBFA2T3	EXT2	JAG2	OFD1	SLC34A2	
CBFB	EYA1	JAK1	OLIG1	SLC45A3	
CBL	EYA2	JAK2	OLIG2	SLC7A5	
CBLB	EZH2	JAK3	OLR1	SLCO1B3	
CBLC	EZR	JARID2	OMD	SLX4	
CCAR2	FAF1	JAZF1	P2RY8	SMAD2	
CCDC28A	FAM127C	JUN	PAFAH1B2	SMAD3	
CCDC6	FAM19A2	KALRN	PAG1	SMAD4	
CCDC88C	FAM19A5	KANK1	PAK1	SMAD6	
CCK	FAM46C	KAT2B	PAK3	SMAP1	
CCL2	FAM64A	KAT6A	PAK6	SMARCA1	
CCNA2	FANCA	KAT6B	PAK7	SMARCA4	
CCNB1IP1	FANCB	KCNB1	PALB2	SMARCA5	
CCNB3	FANCC	KDM1A	PAPPA	SMARCB1	
CCND1	FANCD2	KDM2B	PASK	SMC1A	
CCND2	FANCE	KDM4C	PATZ1	SMC3	
CCND3	FANCF	KDM5A	PAX3	SMO	
CCNE1	FANCG	KDM5C	PAX5	SNAPC3	
CCNG1	FANCI	KDM6A	PAX7	SNCG	
CCT6B	FANCL	KDR	PAX8	SNHG5	

CD19	FANCM	KDSR	PBRM1	SNW1	
CD22	FAS	KEAP1	PBX1	SNX29	
CD274	FASLG	KIAA0232	PC	SNX9	
CD28	FBN2	KIAA1524	PCBP1	SOCS1	
CD36	FBXO11	KIAA1549	PCLO	SOCS2	
CD44	FBXO31	KIAA1598	PCM1	SOCS3	
CD58	FBXW7	KIF5B	PCNA	SOD2	
CD70	FCGBP	KIT	PCNA-AS1	SORBS2	
CD74	FCGR2B	KLF4	PCSK7	SORT1	
CD79A	FCRL4	KLHL6	PDCD1	SOS1	
CD79B	FEN1	KLK2	PDCD11	SOX10	
CD8A	FEV	KLK7	PDCD1LG2	SOX11	
CDC14A	FGF1	KMT2A	PDE4DIP	SOX2	
CDC14B	FGF10	KMT2B	PDGFA	SP1	
CDC25A	FGF13	KMT2C	PDGFB	SP3	
CDC25C	FGF14	KMT2D	PDGFD	SPECC1	
CDC42	FGF19	KPNB1	PDGFRA	SPEN	
CDC73	FGF2	KRAS	PDGFRB	SPOP	
CDH1	FGF23	KSR1	PDK1	SPP1	
CDH11	FGF3	KTN1	PEG3	SPRY2	
CDK1	FGF4	LAMA1	PER1	SPRY4	
CDK12	FGF6	LAMA5	PFDN5	SPTAN1	
CDK2	FGF8	LAMP2	PHB	SPTBN1	
CDK4	FGF9	LASP1	PHF1	SQSTM1	
CDK5RAP2	FGFR1	LCK	PHF23	SRC	
CDK6	FGFR1OP	LCP1	PHF6	SRF	
CDK7	FGFR1OP2	LEF1	PHOX2B	SRGAP3	
CDK8	FGFR2	LEFTY2	PI4KA	SRRM3	
CDK9	FGFR3	LFNG	PICALM	SRSF2	
CDKL5	FGFR4	LGALS3	PIK3CA	SRSF3	
CDKN1A	FH	LGR5	PIK3CB	SS18	
CDKN1B	FHIT	LHFP	PIK3CD	SS18L1	
CDKN1C	FHL2	LHX2	PIK3CG	SSBP2	
CDKN2A	FIGF	LHX4	PIK3R1	SSX1	
CDKN2B	FIP1L1	LIFR	PIK3R2	SSX2	
CDKN2C	FLCN	LINC00598	PIM1	SSX2B	
CDKN2D	FLI1	LINC00982	PKM	SSX4	
CDX1	FLNA	LINGO2	PLA2G2A	SSX4B	
CDX2	FLNC	LMBRD1	PLA2G5	ST6GAL1	
CEBPA	FLT1	LMO1	PLAG1	STAG2	
CEBPB	FLT3	LMO2	PLAT	STAT1	
CEBPD	FLT3LG	LMO7	PLAU	STAT3	
CEBPE	FLT4	LNP1	PLCB1	STAT4	
CENPF	FLYWCH1	LOX	PLCB4	STAT5A	
CENPU	FNBP1	LPAR1	PLCG1	STAT5B	
CEP170B	FOS	LPP	PLCG2	STAT6	

CEP57	FOSB	LPXN	PLEKHM2	STIL	
CEP85L	FOSL1	LRIG3	PML	STK11	
CHCHD7	FOXL2	LRMP	PMS1	STL	
CHD2	FOXO1	LRP1B	PMS2	STRN	
CHD6	FOXO3	LRP5	POFUT1	STX5	
CHEK1	FOXO4	LRPPRC	POLD1	STYK1	
CHEK2	FOXP1	LRRC37B	POLD4	SUFU	
CHIC2	FRK	LRRC59	POLR2H	SUGP2	
CHL1	FRMPD4	LRRC7	POM121	SULF1	
CHMP2B	FRS2	LRRK2	POMGNT1	SUV39H2	
CHN1	FRYL	LTBP1	POSTN	SUZ12	
CHST11	FSTL3	LYL1	POT1	SYK	
CHUK	FUS	LYN	POU2AF1	SYP	
CIC	FUT1	MACROD1	POU5F1	TACC1	
CIITA	FZD10	MAD2L1	PPAP2B	TACC2	
CIRH1A	FZD2	MADD	PPARG	TACC3	
CIT	FZD3	MAF	PPARGC1A	TAF1	
CKB	FZD6	MAFB	PPFIA2	TAF15	
CKS1B	FZD7	MAGED1	PPFIBP1	TAL1	
CLP1	FZD8	MAGEE1	PPM1D	TAL2	
CLTA	GAB1	MALAT1	PPP1CB	TAOK1	
CLTC	GABRG2	MALT1	PPP1R13B	TBL1XR1	
CLTCL1	GADD45B	MAML1	PPP1R13L	TBX15	
CMKLR1	GANAB	MAML2	PPP2CB	TCEA1	

Shown are the 1408 genes tested in patient samples before and/or after transplantation.

Table S4a: *Patient and Donor Demographics*

Subject No	Sample Timing[a]	Test vs Validation[b]	Subject Gender[c]	Diagnosis[d]	Subject Age	Donor Type	HLA Match	Donor Gender[c]	Donor Age
1	Post	T	M	AML	49	Unrelated	Match	M	30
2	Both	T	M	MDS	65	Unrelated	Match	M	20
3	Pre	T	F	MDS	73	Related	Haplo	F	49
4	Post	T	M	AML	57	Related	Match	F	59
5	Post	T	F	AML	66	Related	Haplo	M	24
6	Post	T	M	AML	54	Related	Haplo	M	18
7	Pre	T	M	MDS	58	Related	Haplo	M	23
8	Post	T	F	ALL	63	Unrelated	Match	M	28
9	Pre	T	M	MPD	52	Unrelated	9/10	F	23
10	Both	T	M	MDS	66	Related	Haplo	F	26
11	Post	T	F	MDS	71	Unrelated	Match	M	28
12	Post	T	M	MDS	71	Unrelated	Match	F	24
13	Post	T	F	MPD	60	Unrelated	Match	F	24
14	Post	T	F	AML	67	Unrelated	9/10	M	44
15	Post	T	M	MDS	67	Unrelated	Match	M	27
16	Pre	T	M	MPD	62	Related	Haplo	M	35
17	Both	T	F	AML	63	Unrelated	Match	M	30
18	Both	T	F	MDS	25	Unrelated	Match	F	31
19	Post	T	M	ALL	61	Unrelated	Match	M	18
20	Post	T	F	MDS	62	Unrelated	9/10	F	54
21	Pre	T	M	MPD	70	Unrelated	Match	M	23
22	Pre	T	F	ALL	59	Unrelated	9/10	M	39
23	Both	T	M	MDS	71	Unrelated	Match	M	21
24	Both	T	F	ALL	70	Unrelated	Match	F	25
25	Post	T	F	ALL	58	Unrelated	Match	M	31
26	Both	T	M	MPD	74	Unrelated	Match	M	28
27	Pre	T	M	AML	59	Unrelated	Match	F	23
28	Pre	T	M	MPD	62	Related	Match	M	59
29	Both	T	F	AML	71	Unrelated	Match	F	22

30	Post	T	F	AML	61	Unrelated	Match	M	42
31	Both	T	M	MDS	63	Related	Match	F	57
32	Pre	T	F	AML	68	Related	Haplo	M	25
33	Both	T	F	AML	37	Unrelated	8/10	M	21
34	Both	T	F	MDS	55	Unrelated	Match	M	33
35	Pre	T	M	AML	68	Unrelated	9/10	M	28
36	Pre	T	M	AML	64	Unrelated	Match	M	32
37	Pre	T	F	MPD	73	Unrelated	Match	M	31
38	Pre	T	M	MDS	72	Unrelated	Match	M	38
39	Pre	T	M	MDS	26	Unrelated	Match	M	19
40	Both	T	F	MDS	64	Related	Haplo	M	22
41	Post	T	F	AML	26	Related	Match	M	29
42	Pre	T	F	MPD	68	Related	Match	F	64
43	Post	T	F	AML	45	Related	Haplo	M	38
44	Post	T	M	AML	73	Unrelated	Match	M	29
45	Both	T	F	AML	56	Unrelated	Match	M	38
46	Post	T	F	MDS	76	Unrelated	Match	M	27
47	Post	T	M	MDS	38	Unrelated	9/10	M	22
48	Post	T	F	AML	72	Unrelated	Match	F	25
49	Both	T	M	AML	65	Unrelated	9/10	M	21
50	Both	T	M	MDS	68	Unrelated	Match	M	18
51	Pre	T	M	MDS	77	Unrelated	Match	M	20
52	Pre	T	F	MDS	64	Related	Haplo	F	36
53	Pre	T	M	NHL	51	Related	Match	M	39
54	Both	T	F	MDS	66	Unrelated	Match	F	21
55	Both	T	F	ALL	54	Related	Haplo	M	26
56	Pre	T	F	AML	60	Related	Haplo	M	32
57	Pre	T	M	MDS	68	Unrelated	Match	M	29
58	Pre	T	M	MDS	71	Related	Haplo	M	42
59	Post	T	F	AML	53	Unrelated	Match	M	18
60	Pre	T	F	NHL	62	Unrelated	Match	F	33
61	Both	T	M	MDS	76	Unrelated	Match	M	18
62	Post	T	M	AML	58	Unrelated	Match	M	34

63	Both	T	M	MPD	59	Unrelated	Match	M	40
64	Pre	T	F	MDS	57	Related	Haplo	M	23
65	Both	T	F	CML	59	Unrelated	9/10	M	21
66	Post	T	M	ALL	64	Unrelated	Match	F	38
67	Pre	T	M	MDS	55	Related	Haplo	F	24
68	Both	T	F	MPD	67	Unrelated	Match	F	28
69	Post	T	F	AML	48	Unrelated	Match	M	50
70	Pre	T	F	MPD	57	Related	Haplo	F	55
71	Both	T	M	MDS	71	Related	Haplo	M	29
72	Both	T	F	AML	72	Related	Haplo	M	40
73	Both	T	M	MDS	70	Related	Haplo	F	41
74	Pre	T	F	MDS	70	Unrelated	Match	M	23
75	Post	T	M	AML	52	Related	Match	F	46
76	Post	T	M	CML	35	Related	Haplo	M	14
77	Post	T	M	MPD	63	Unrelated	Match	M	25
78	Post	T	M	MPD	38	Related	Haplo	F	35
79	Both	T	F	ALL	63	Unrelated	Match	M	22
80	Pre	T	M	NHL	34	Unrelated	Match	M	26
81	Post	T	F	AML	49	Related	Haplo	M	48
82	Post	T	M	ALL	26	Related	Haplo	M	56
83	Both	T	F	AML	64	Unrelated	9/10	M	29
84	Both	T	M	AML	46	Related	Match	F	44
85	Pre	T	F	MPD	49	Related	Match	F	47
86	Both	T	F	AML	39	Related	Haplo	F	18
87	Post	T	M	MDS	69	Unrelated	9/10	M	45
88	Post	T	F	MPD	67	Related	Haplo	M	39
89	Post	T	M	AML	28	Unrelated	Match	M	34
90	Post	T	M	AML	35	Unrelated	Match	M	34
91	Pre	T	M	AML	67	Unrelated	9/10	M	32
92	Both	T	M	AML	70	Related	Haplo	F	36
93	Both	T	M	ALL	56	Related	Haplo	F	55
94	Both	T	F	MPD	72	Unrelated	Match	M	27
95	Both	T	M	AML	68	Related	Haplo	F	21

96	Pre	T	M	AML	66	Unrelated	Match	M	27
97	Pre	T	M	ALL	34	Unrelated	Match	F	32
98	Both	T	M	ALL	30	Unrelated	Match	F	36
99	Pre	T	F	MPD	49	Unrelated	Match	M	26
100	Pre	T	F	MPD	66	Related	Haplo	M	28
101	Pre	T	M	AML	61	Unrelated	Match	F	31
102	Both	T	F	CML	29	Related	Haplo	F	26
103	Both	T	M	MDS	60	Unrelated	Match	M	21
104	Pre	T	M	MPD	67	Related	Haplo	M	39
105	Post	T	F	MPD	76	Unrelated	Match	M	20
106	Both	T	F	MPD	69	Unrelated	Match	M	21
107	Both	T	M	AML	45	Related	Haplo	M	43
108	Both	T	F	AML	69	Unrelated	Match	M	26
109	Pre	T	M	MPD	75	Unrelated	Match	M	27
110	Both	T	M	MDS	64	Related	Haplo	M	30
111	Pre	T	M	AML	41	Related	Haplo	M	37
112	Pre	T	M	ALL	62	Related	Haplo	F	22
113	Both	T	F	AML	66	Unrelated	Match	F	25
114	Pre	T	F	MPD	65	Related	Haplo	M	31
115	Both	T	M	SAA	36	Related	Match	M	32
116	Both	T	F	AML	44	Related	Match	F	22
117	Both	T	M	MPD	79	Unrelated	Match	M	32
118	Pre	T	F	ALL	42	Related	Haplo	M	44
119	Pre	T	M	ALL	36	Related	Haplo	F	28
120	Pre	T	M	MDS	57	Unrelated	Match	M	38
121	Both	T	M	NHL	25	Unrelated	Match	F	33
122	Both	T	F	MDS	64	Unrelated	Match	M	28
123	Pre	T	F	AML	73	Related	Haplo	M	45
124	Pre	T	F	MDS	66	Unrelated	Match	M	30
125	Pre	T	M	AML	48	Related	Haplo	M	19
126	Both	T	M	AML	68	Unrelated	Match	F	22
127	Both	T	F	MPD	74	Unrelated	Match	M	29
128	Both	T	F	MDS	67	Related	Haplo	M	35

129	Pre	T	F	NHL	62	Related	Haplo	M	40
130	Both	T	M	AML	56	Unrelated	Match	M	30
131	Both	V	M	AML	64.2	Related	Haplo	M	22
132	Both	V	M	MDS	32.7	Unrelated	8/10	M	22
133	Both	V	M	ALL	30.2	Related	Match	M	32
134	Both	V	F	NHL	22.9	Unrelated	9/10	F	27
135	Both	V	M	MDS	64.7	Unrelated	Match	F	19
136	Both	V	F	AML	63.7	Unrelated	Match	F	27
137	Both	V	F	MDS	69.8	Unrelated	9/10	M	36
138	Both	V	M	MDS	73.9	Related	Haplo	F	23
139	Both	V	M	AML	49.0	Unrelated	Match	M	23
140	Both	V	F	MPD	70.0	Unrelated	Match	M	23
141	Both	V	M	MDS	70.5	Related	Haplo	F	39
142	Both	V	M	MPD	64.0	Unrelated	Match	F	21
143	Both	V	F	ALL	26.7	Unrelated	Match	F	20
144	Both	V	M	MPD	60.5	Unrelated	Match	M	24
145	Both	V	F	AML	68.4	Unrelated	Match	M	31
146	Both	V	M	AML	78.4	Unrelated	Match	F	20
147	Both	V	F	AML	62.4	Related	Haplo	F	33
148	Both	V	F	MDS	51.2	Related	Haplo	F	38
149	Both	V	M	ALL	64.0	Related	Haplo	M	34
150	Both	V	M	MDS	64.2	Unrelated	Match	M	20
151	Both	V	F	AML	31.3	Related	Haplo	F	27
152	Both	V	F	AML	28.9	Unrelated	Match	M	26
153	Pre	V	F	SAA	20.8	Related	Match	F	22
154	Both	V	M	MPD	72.7	Unrelated	Match	M	28
155	Both	V	M	MPD	65.3	Related	Haplo	M	29
156	Pre	V	F	SAA	62.3	Unrelated	Match	F	19
157	Both	V	M	NHL	66.8	Unrelated	Match	M	33
158	Both	V	F	AML	74.3	Unrelated	Match	F	29
159	Both	V	M	ALL	48.7	Unrelated	Match	M	25
160	Both	V	F	AML	48.3	Related	Match	F	52
161	Pre	V	F	SAA	44.5	Related	Haplo	F	20

162	Both	V	M	MPD	72.6	Unrelated	Match	M	24
163	Both	V	M	MDS	65.4	Unrelated	9/10	F	21
164	Both	V	F	ALL	47.4	Unrelated	Match	F	35
165	Both	V	M	AML	32.8	Unrelated	Match	M	29
166	Both	V	M	AML	31.1	Unrelated	9/10	M	24
167	Both	V	F	MPD	64.2	Related	Haplo	M	35

Shown are patient and donor characteristics for all 167 subjects enrolled into study.

[a] Samples obtained pre-transplant (Pre), post-transplant (Post), or pre- and post-transplant (Both).

[b] Samples included in either the test (T) or validation (V) set.

[c] M=male sex; F=female sex.

[d] Definitions of diagnosis are provided in Table 1 of the manuscript.

Table S4b: *Transplant Source and Conditioning and GvHD Regimens*

Subject No	HSC Source[a]	Conditioning Regimen[b]	Conditioning Intensity[b]	GVHD Prophylaxis[b]	Abatacept Y/N[c]	ATG Y/N[c]
1	PBSC	FluBu4	MA	TacMTX	N	Y
2	PBSC	FluMel100	RIC	TacMTX	N	Y
3	Marrow	FluCyTBI	NMA	PTCy	N	N
4	PBSC	TBICy	MA	TacMTX	N	N
5	PBSC	FluMelTBI	RIC	PTCy	Y	N
6	Marrow	FluTBI1200	MA	PTCy	N	N
7	PBSC	FluCyTBI	NMA	PTCy	Y	N
8	PBSC	FluMel100	RIC	TacMTX	N	Y
9	PBSC	FluMelTBI	RIC	PTCy	Y	N
10	PBSC	FluCyTBI	NMA	PTCy	N	N
11	PBSC	FluBu2	RIC	TacMTX	N	Y
12	PBSC	FluBu2	RIC	TacMTX	N	Y
13	PBSC	FluMel100	RIC	TacMTX	N	Y
14	PBSC	FluCyTBI	NMA	PTCy	N	N
15	PBSC	FluBu2	RIC	TacMTX	N	Y
16	Marrow	FluCyTBI	NMA	PTCy	N	N
17	PBSC	FluBu4	MA	TacMTX	N	Y
18	PBSC	FluMelTBI	RIC	PTCy	Y	N
19	PBSC	FluMel	RIC	TacMTX	N	Y
20	PBSC	FluCyTBI	NMA	PTCy	N	N
21	PBSC	FluBu2	RIC	TacMTX	N	Y
22	Marrow	FluMelTBI	RIC	PTCy	N	N
23	PBSC	FluBu2	RIC	TacMTX	N	Y
24	PBSC	FluMel100	RIC	TacMTX	N	Y
25	PBSC	FluMel140	RIC	TacMTX	N	Y
26	PBSC	FluBu2	RIC	TacMMFRapa	N	Y
27	PBSC	FluBu4	MA	TacMTX	N	Y
28	PBSC	FluTBI300	NMA	RapaCSPMMF	N	N

29	PBSC	FluBu2	RIC	TacMTX	N	Y
30	PBSC	FluMel140	RIC	TacMTX	N	Y
31	PBSC	FluBu4	MA	TacMTX	N	N
32	PBSC	FluCyTBI	NMA	PTCy	N	N
33	PBSC	FluMel100TBI	RIC	PTCy	Y	N
34	PBSC	FluMel100	RIC	TacMTX	N	Y
35	PBSC	FluMel140	RIC	TacMTX	N	Y
36	PBSC	FluMel100	RIC	TacMTX	N	Y
37	PBSC	FluTBI300	NMA	RapaCSPMMF	N	N
38	PBSC	FluTBI300	NMA	RapaCSPMMF	N	N
39	PBSC	FluBu4	MA	TacMTX	N	N
40	PBSC	FluCyTBI	NMA	PTCy	N	N
41	PBSC	FluBu4	MA	TacMTX	N	N
42	PBSC	FluBu2	RIC	TacMTX	N	Y
43	PBSC	FluMelTBI	RIC	PTCy	Y	N
44	PBSC	FluBu2	RIC	TacMTX	N	Y
45	PBSC	FluMel100TBI	RIC	TacMTX	N	N
46	PBSC	FluTBI300	NMA	RapaCSPMMF	N	N
47	Marrow	FluTBI1200	MA	PTCy	N	N
48	PBSC	FluTBI300	NMA	RapaCSPMMF	N	N
49	PBSC	FluMelTBI	RIC	PTCy	N	N
50	PBSC	FluBu2	RIC	TacMTX	N	Y
51	PBSC	FluTBI300	NMA	RapaCSPMMF	N	N
52	Marrow	FluMelTBI	RIC	PTCy	N	N
53	PBSC	FluMelThio	RIC	TacMTX	N	N
54	PBSC	FluTBI300	NMA	RapaCSPMMF	N	N
55	Marrow	FluMelTBI	RIC	PTCy	N	N
56	PBSC	FluCyTBI	NMA	PTCy	N	N
57	PBSC	FluMel100	RIC	TacMTX	N	Y
58	Marrow	FluMelTBI	RIC	PTCy	N	N
59	Marrow	FluMel140	RIC	TacMTX	N	Y
60	PBSC	FluMelTBI	RIC	PTCy	Y	N
61	PBSC	FluTBI300	NMA	RapaCSPMMF	N	N

62	PBSC	FluBu4	MA	TacMTX	N	Y
63	PBSC	FluMel140	RIC	TacMTX	N	Y
64	Marrow	FluCyTBI	NMA	PTCy	N	N
65	Marrow	FluMelTBI	RIC	PTCy	N	N
66	PBSC	FluTBI300	NMA	RapaCSPMMF	N	N
67	Marrow	FluMel100TBI	RIC	PTCy	N	N
68	PBSC	FluMel140	RIC	TacMTX	N	Y
69	PBSC	FluBu4	MA	TacMTX	N	Y
70	PBSC	FluCyTBI	NMA	PTCy	Y	N
71	PBSC	FluCyTBI	NMA	PTCy	Y	N
72	PBSC	FluCyTBI	NMA	PTCy	N	N
73	Marrow	FluCyTBI	NMA	PTCy	N	N
74	PBSC	FluBu2	RIC	TacMTX	N	Y
75	PBSC	FluBu4	MA	TacMTX	N	N
76	PBSC	FluTBI1200	MA	PTCy	N	N
77	PBSC	FluMel140	RIC	TacMTX	N	Y
78	Marrow	FluTBI1200	MA	PTCy	Y	N
79	Marrow	FluMel140	RIC	TacMTX	N	Y
80	PBSC	TBICy	MA	TacMTX	N	N
81	Marrow	FluMel100TBI	RIC	PTCy	N	N
82	Marrow	FluTBI1200	MA	PTCy	N	N
83	PBSC	FluTBI300	NMA	RapaCSPMMF	Y	N
84	Marrow	FluBu4	MA	TacMTX	N	N
85	PBSC	FluBu4	MA	TacMTX	N	N
86	Marrow	FluTBI1200	MA	PTCy	N	N
87	PBSC	FluTBI300	NMA	RapaCSPMMF	N	N
88	Marrow	FluMel100TBI	RIC	PTCy	N	N
89	PBSC	FluBu4	MA	TacMTX	Y	Y
90	PBSC	FluBu4	MA	TacMTX	Y	Y
91	PBSC	FluMel100TBI	RIC	PTCy	N	N
92	PBSC	FluCyTBI	NMA	PTCy	N	N
93	PBSC	FluCyTBI	NMA	PTCy	N	N
94	PBSC	FluBu2	RIC	TacMTX	N	Y

95	Marrow	FluCyTBI	NMA	PTCy	N	N
96	PBSC	FluBu4	MA	TacMTX	N	Y
97	Marrow	TBICy	MA	TacMTX	N	N
98	PBSC	TBICy	MA	TacMTX	N	N
99	PBSC	FluBu2	RIC	TacMTX	Y	Y
100	Marrow	FluMel100TBI	RIC	PTCy	N	N
101	PBSC	FluTBI300	NMA	RapaCSPMMF	N	N
102	Marrow	FluTBI1200	MA	PTCy	N	N
103	PBSC	FluMel140	RIC	TacMTX	N	Y
104	PBSC	FluCyTBI	NMA	PTCy	N	N
105	PBSC	FluBu2	RIC	TacMTX	N	Y
106	PBSC	FluBu2	RIC	TacMTX	N	Y
107	Marrow	FluTBI1200	MA	PTCy	N	N
108	PBSC	FluBu2	RIC	TacMTX	N	Y
109	PBSC	FluTBI300	NMA	RapaCSPMMF	N	N
110	Marrow	FluMel100TBI	RIC	PTCy	N	N
111	PBSC	FluTBI1200	MA	PTCy	N	N
112	Marrow	FluMel100TBI	RIC	PTCy	N	N
113	PBSC	FluMel140	RIC	TacMTX	N	Y
114	Marrow	FluMel100TBI	RIC	PTCy	N	N
115	Marrow	CyATG	NMA	TacMTX	N	Y
116	PBSC	FluBu4	MA	TacMTX	N	N
117	PBSC	FluTBI300	NMA	RapaCSPMMF	N	N
118	Marrow	FluTBI1200	MA	PTCy	N	N
119	Marrow	FluTBI1200	MA	PTCy	N	N
120	PBSC	FluBu4	MA	TacMTX	Y	Y
121	PBSC	FluMel100TBI	RIC	PTCy	N	N
122	PBSC	FluMel100	RIC	TacMTX	N	Y
123	PBSC	FluMel100TBI	RIC	PTCy	Y	N
124	PBSC	FluTBI300	NMA	RapaCSPMMF	N	N
125	PBSC	FluMel100TBI	RIC	PTCy	Y	N
126	PBSC	FluMel100	RIC	TacMTX	N	Y
127	PBSC	FluTBI300	NMA	RapaCSPMMF	N	N

128	PBSC	FluCyTBI	NMA	PTCy	N	N
129	PBSC	FluMel100TBI	RIC	PTCy	Y	N
130	PBSC	FluBu4	MA	TacMTX	N	Y
131	PBSC	FluMelTBI	RIC	PTCy	N	N
132	PBSC	FluCyTBI	NMA	PTCy	Y	N
133	Marrow	TBICy	MA	TacMTX	N	N
134	PBSC	FluMelTBI	RIC	PTCy	Y	N
135	PBSC	FluMel	RIC	TacMTX	Y	Y
136	PBSC	FluBu4	MA	TacMTX	N	Y
137	PBSC	FluCyTBI	NMA	PTCy	Y	N
138	PBSC	FluCyTBI	NMA	PTCy	N	N
139	PBSC	FluMel	RIC	TacMTX	Y	Y
140	PBSC	FluBu2	RIC	TacMTX	Y	Y
141	PBSC	FluCyTBI	NMA	PTCy	N	N
142	PBSC	FluMel	RIC	TacMTX	Y	Y
143	PBSC	FluTBI1200	MA	PTCy	N	N
144	PBSC	FluBu2	RIC	TacMTX	Y	Y
145	PBSC	FluBu2	RIC	TacMTX	N	Y
146	PBSC	FluTBI300	NMA	RapaCSPMMF	N	N
147	PBSC	FluCyTBI	NMA	PTCy	Y	N
148	PBSC	FluTBI1200	MA	PTCy	N	N
149	Marrow	FluMelTBI	RIC	PTCy	Y	N
150	PBSC	FluTBI300	NMA	RapaCSPMMF	N	N
151	Marrow	FluTBI1200	MA	PTCy	N	N
152	PBSC	FluTBI1200	MA	PTCy	N	N
153	Marrow	CyATG	NMA	TacMTX	N	Y
154	Marrow	FluMel	RIC	TacMTX	N	N
155	PBSC	FluCyTBI	NMA	PTCy	N	N
156	PBSC	FluBu2	RIC	TacMTX	Y	Y
157	PBSC	FluTBI300	NMA	RapaCSPMMF	N	N
158	PBSC	FluMel	RIC	TacMTX	Y	Y
159	PBSC	FluTBI1200	MA	PTCy	Y	N
160	PBSC	FluBu4	MA	TacMTX	N	N

161	PBSC	FluCyTBI	NMA	PTCy	Y	N
162	PBSC	FluBu2	RIC	TacMtx	N	Y
163	PBSC	FluMelTBI	RIC	PTCy	Y	N
164	PBSC	FluTBI1200	MA	PTCy	Y	N
165	PBSC	FluBu4	MA	TacMTX	Y	Y
166	Marrow	FluTBI1200	MA	PTCy	N	N
167	PBSC	FluMelTBI	RIC	PTCy	N	N

Shown are graft source, conditioning regimen, and GvHD prophylaxis regimens for all 167 subjects enrolled into study.

[a] HSC=hematopoietic stem cell; PBSC=peripheral blood stem cells; Marrow= bone marrow

[b] Definitions of conditioning and GvHD regimens are provided in Tables S1 and S2 of the supplement.

[c] Y=yes; N=no

Table S4c: *aGvHD and Survival*

Subject No	Relapse after Transplant[a]	Days to Relapse	Day DLI Given[b]	aGVHD (Y/N)[b]	aGvHD Evaluable	Day Onset aGvHD	aGvhd Skin	aGvhd Liver	aGvHD UGI[b]	aGvhd LGI[b]	aGvHD Max Stage	Deceased	Days Follow-up or Death
1	N		NA	Y	Y	21	3	0	0	0	2	N	490
2	N		NA	Y	Y	36	3	0	0	0	2	N	387
3	Y	64	135	Y	N	154	0	4	0	0	4	Y	161
4	N		NA	N	Y	NA	0	0	0	0	0	N	637
5	N		NA	Y	Y	28	0	0	1	0	2	N	359
6	N		NA	Y	Y	62	0	0	1	2	3	N	721
7	N		NA	N	N	NA	0	4	0	0	UNK	Y	22
8	N		NA	Y	Y	28	2	0	1	2	2	N	344
9	N		NA	Y	Y	18	3	0	0	2	3	N	283
10	N		NA	Y	Y	27	3	0	1	0	2	Y	515
11	N		NA	N	Y	NA	0	0	0	0	0	N	824
12	N		NA	Y	Y	40	2	0	0	0	1	N	814
13	Y	718	803	N	Y	NA	0	0	0	0	0	N	803
14	N		NA	N	Y	NA	0	0	0	0	0	Y	166
15	N		NA	Y	Y	99	1	0	1	0	2	N	725
16	N		NA	UNK	Y	NA	0	0	0	0	0	Y	24
17	N		NA	Y	Y	37	3	0	0	0	2	N	562
18	N		NA	Y	Y	15	0	0	1	0	2	N	282
19	N		NA	Y	Y	85	3	0	0	0	2	N	782
20	N		NA	Y	Y	17	0	0	1	0	2	N	794
21	N		NA	N	Y	NA	0	0	0	0	0	N	664
22	N		NA	Y	Y	37	0	0	1	0	2	N	371
23	N		NA	N	Y	NA	0	0	0	0	0	N	547
24	N		329	Y	Y	19	0	0	1	0	2	N	463
25	N		NA	Y	Y	45	2	0	1	0	0	N	720
26	N		NA	N	Y	NA	0	0	0	0	0	Y	39
27	Y	35	NA	Y	Y	31	0	0	1	0	2	Y	74
28	Y	104	140	Y	N	192	0	3	0	4	4	Y	239
29	N		111	Y	N	161	2	0	1	0	2	Y	358

30	N		160	N	Y	NA	0	0	0	0	0	N	608
31	N		NA	Y	Y	15	0	0	1	0	2	N	777
32	Y	92	107	Y	Y	19	0	0	1	1	2	Y	209
33	N		NA	Y	Y	21	1	0	1	0	2	Y	242
34	N		NA	Y	Y	33	0	0	1	0	2	Y	154
35	N		NA	Y	Y	22	0	0	1	0	2	Y	59
36	N		207	Y	Y	26	3	0	0	0	2	N	446
37	N		NA	Y	Y	125	2	0	0	0	1	Y	560
38	Y	64	153	N	Y	NA	0	0	0	0	0	Y	236
39	Y	98	146	Y	Y	32	3	0	0	0	2	N	591
40	N		NA	Y	Y	21	3	0	0	0	2	N	297
41	N		NA	N	Y	49	1	0	0	0	1	N	617
42	N		NA	Y	Y	35	2	0	0	0	1	N	758
43	N		NA	Y	Y	49	0	0	0	1	2	N	817
44	N		102	Y	N	130	3	0	0	0	2	UNK	304
45	N		NA	Y	Y	50	2	0	0	0	1	N	408
46	N		NA	Y	Y	120	2	0	0	0	1	N	869
47	Y	329	348	Y	Y	25	1	0	0	0	1	N	597
48	N		NA	Y	Y	67	1	0	0	0	1	N	432
49	N		NA	N	Y	NA	0	0	0	0	0	Y	243
50	Y	81	NA	Y	Y	55	3	0	0	0	2	Y	161
51	N		NA	Y	Y	11	3	0	0	0	2	N	562
52	N		NA	Y	Y	38	3	0	0	4	4	Y	204
53	N		NA	N	Y	NA	0	0	0	0	0	Y	26
54	Y	84	NA	N	Y	NA	0	0	0	0	0	Y	142
55	N		NA	N	Y	NA	0	0	0	0	0	N	820
56	N		NA	N	Y	NA	0	0	0	0	0	Y	7
57	N		NA	N	Y	NA	0	0	0	0	0	Y	17
58	N		NA	Y	Y	46	0	0	1	0	2	Y	79
59	N		NA	Y	Y	22	0	0	0	1	2	N	800
60	N		NA	Y	Y	26	3	0	1	0	2	Y	238
61	Y	93	148	N	Y	NA	0	0	0	0	0	N	419
62	Y	74	104	Y	Y	18	0	0	1	1	2	Y	120

63	Y	87	NA	N	Y	NA	0	0	0	0	0	N	430
64	N		NA	N	Y	NA	0	0	0	0	0	Y	37
65	N		179	Y	Y	44	2	0	0	0	2	N	279
66	N		NA	Y	Y	104	0	0	1	0	2	N	731
67	Y	222	167	Y	N	226	0	0	0	1	2	Y	309
68	N		172	N	Y	NA	0	0	0	0	0	N	685
69	N		NA	Y	Y	17	3	1	1	0	2	N	602
70	N		NA	Y	Y	66	3	0	0	0	2	Y	383
71	Y	88	NA	Y	Y	72	3	0	0	1	2	Y	179
72	N		NA	Y	Y	55	3	0	0	0	2	Y	165
73	N		NA	Y	Y	46	3	0	0	0	2	N	366
74	Y	48	NA	N	Y	49	0	0	1	0	2	Y	59
75	N		NA	N	Y	NA	0	0	0	0	0	Y	509
76	N		NA	Y	Y	41	2	0	0	0	1	N	683
77	N		NA	Y	Y	32	2	0	0	0	1	N	925
78	N		NA	Y	Y	67	0	0	1	0	2	N	849
79	N		NA	Y	Y	43	2	0	0	0	1	N	786
80	N		NA	Y	Y	10	2	2	0	4	3	Y	79
81	N		NA	Y	Y	47	0	0	1	0	2	N	822
82	Y	80	NA	N	Y	NA	0	0	0	0	0	Y	155
83	Y	32	78	Y	N	119	3	0	0	0	2	Y	175
84	N		NA	N	Y	NA	0	0	0	0	0	N	205
85	N		146	N	Y	NA	0	0	0	0	0	N	684
86	N		NA	Y	Y	27	2	0	0	0	1	N	714
87	Y	133	92	N	Y	NA	0	0	0	0	0	Y	211
88	N		NA	N	Y	NA	0	0	0	0	0	N	733
89	N		NA	N	Y	NA	0	0	0	0	0	N	842
90	N		NA	N	Y	NA	0	0	0	0	0	N	744
91	N		NA	Y	Y	40	3	0	0	0	2	Y	86
92	Y	118	NA	Y	Y	32	1	0	1	0	1	N	191
93	N		NA	Y	Y	31	3	0	0	0	2	N	772
94	N		NA	Y	Y	30	0	0	1	0	2	N	766
95	Y	332	NA	Y	Y	49	3	0	1	0	2	Y	384

96	N		NA	Y	Y	24	2	0	0	0	1	N	755
97	N		NA	Y	Y	55	0	0	0	1	2	Y	147
98	N		NA	Y	Y	31	0	0	1	0	2	Y	459
99	N		NA	UNK	Y	NA	0	0	0	0	0	Y	45
100	N		NA	Y	Y	86	1	0	0	0	1	N	641
101	N		NA	N	Y	NA	0	0	0	0	0	N	548
102	N		NA	Y	Y	48	1	0	0	0	1	N	574
103	N		NA	Y	Y	13	0	0	1	0	2	N	374
104	N		NA	N	Y	NA	0	0	0	0	0	Y	89
105	N		NA	Y	Y	92	0	0	0	1	2	N	632
106	N		429	Y	Y	88	0	0	1	0	2	N	620
107	N		NA	Y	Y	60	0	0	1	0	2	Y	327
108	N		NA	Y	Y	26	3	0	1	0	2	N	616
109	N		NA	Y	Y	93	3	0	0	2	3	Y	117
110	N		NA	Y	Y	20	3	0	1	0	2	N	551
111	N		NA	Y	Y	45	3	0	0	0	2	N	564
112	Y	171	NA	N	Y	NA	0	0	0	0	0	N	298
113	Y	157	NA	Y	Y	43	2	0	0	0	1	Y	170
114	N		NA	N	Y	NA	0	0	0	0	0	N	426
115	N		NA	N	Y	NA	0	0	0	0	0	N	466
116	N		NA	N	Y	NA	0	0	0	0	0	N	436
117	N		NA	N	Y	NA	0	0	0	0	0	N	312
118	N		NA	Y	Y	27	0	0	1	1	2	Y	135
119	Y	154	NA	Y	Y	90	1	0	0	0	1	Y	168
120	Y	187	NA	Y	Y	47	0	0	1	0	2	N	275
121	N		NA	N	Y	NA	0	0	0	0	0	N	232
122	N		NA	Y	Y	33	3	0	1	0	2	N	359
123	N		NA	Y	Y	26	0	0	1	0	2	Y	310
124	N		NA	Y	Y	40	0	0	1	0	2	N	292
125	N		NA	N	Y	NA	0	0	0	0	0	N	364
126	N		137	N	Y	NA	0	0	0	0	0	N	380
127	N		NA	Y	Y	100	0	0	1	0	2	Y	266
128	N		NA	Y	Y	39	2	0	1	0	2	N	264

129	N		NA	N	Y	NA	0	0	0	0	0	Y	338
130	N		NA	Y	Y	27	2	0	0	0	1	N	294
131	N		NA	Y	Y	57	2	0	0	1	2	N	385
132	NA		NA	Y	Y	110	2	0	0	0	1	N	294
133	NA		NA	N	Y	NA	0	0	0	0	0	N	278
134	NA		NA	Y	Y	69	0	0	1	0	2	N	245
135	N		NA	N	Y	NA	0	0	0	0	0	N	238
136	NA		NA	Y	Y	20	3	0	0	0	2	N	352
137	Y	28	NA	Y	Y	21	0	0	1	0	2	Y	184
138	Y	55	NA	Y	Y	68	0	0	1	0	2	Y	373
139	N		NA	N	Y	NA	0	0	0	0	0	N	182
140	Y	35	NA	Y	Y	47	0	1	1	0	2	N	326
141	Y	82	NA	Y	Y	36	3	0	0	0	2	Y	142
142	Y	166	170	N	Y	NA	0	0	0	0	0	N	266
143	Y	88	NA	N	Y	NA	0	0	0	0	0	Y	131
144	Y	52	NA	Y	Y	120	3	0	0	0	2	Y	151
145	NA		NA	Y	Y	16	2	0	0	0	1	N	414
146	NA		NA	Y	Y	91	2	0	0	0	1	Y	408
147	Y	52	99	Y	Y	24	1	0	0	0	1	Y	125
148	Y	27	NA	Y	Y	53	3	0	0	0	2	N	393
149	N		NA	Y	Y	67	3	0	0	0	2	N	221
150	Y	82	161	Y	Y	35	0	0	1	0	2	Y	198
151	NA		NA	N	Y	NA	0	0	0	0	0	N	372
152	NA		NA	Y	Y	31	3	0	0	0	2	N	292
153	NA		NA	Y	Y	53	0	0	1	0	2	N	413
154	NA		194	Y	Y	18	0	1	0	1	2	N	438
155	Y	92	NA	Y	Y	128	2	0	0	0	1	N	312
156	NA		NA	N	Y	NA	0	0	0	0	0	N	180
157	NA		NA	Y	Y	20	0	0	1	0	2	Y	129
158	N		NA	N	Y	NA	0	0	0	0	0	N	172
159	NA		NA	Y	Y	21	2	0	0	0	1	N	329
160	NA		NA	N	Y	NA	0	0	0	0	0	N	346
161	NA		NA	N	Y	NA	0	0	0	0	0	N	185

162	N		131	N	Y	NA	0	0	0	0	0	N	253
163	NA		NA	Y	Y	34	3	0	1	0	2	N	174
164	NA		NA	N	Y	NA	0	0	0	0	0	N	172
165	N		NA	N	Y	NA	0	0	0	0	0	N	163
166	NA		NA	N	Y	NA	0	0	0	0	0	N	411
167	N		NA	Y	Y	45	0	0	1	0	2	N	392

Shown are outcomes including aGvHD and overall survival for all 167 subjects enrolled into study.

[a] Y=yes; N=no

[b] DLI=donor lymphocyte infusion; aGvHD=acute graft-versus-host disease; UGI=upper GI; LGI=lower GI

Table S5: *400 Genes Predicting aGvHD in Pre-transplant Samples*

1-100	101-200	201-300	301-400
MAFB	UBE2C	BACH2	EEFSEC
CTNNB1	YPEL5	CD274	ELP2
DDX5	AFF1	CDK5RAP2	EML4
AKAP9	CASC5	CSNK2A1	EPS15
RELA	ERCC5	CYLD	FAM19A2
CDKN2D	GANAB	FGF14	FOXO1
FANCM	GOPC	GTSE1	HERPUD1
IRF2BP2	ITGA5	HIST1H2AG	JAK2
MN1	MPL	MAML2	LASP1
PPP2R1B	MTOR	MB21D2	LHFP
SMAD6	MYBL1	MGEA5	LNP1
TP53BP1	NUP107	MYCL	MACROD1
CDKN1B	SF3B1	MYD88	MBNL1
FRS2	SIK3	NCOA3	MBTD1
FRYL	SQSTM1	NDRG1	MLF1
KDM4C	TP53	PASK	NIPBL
MCL1	USP16	PLAT	NUTM2B
43717SEPT9	ZNF384	PLEKHM2	OLIG2
TCF12	ACSL3	PRDM1	PC
TSC1	ALDH2	PRKACA	PCM1
CTCF	ARAF	RAD51B	PIK3R2
GAB1	ASXL1	RPN1	PKM
HIST1H2BJ	CEBPB	SIRT1	PLCG1
ARIH2	FGFR3	TLR4	PPP2R4
GNA13	HIST1H2AC	WHSC1	PPP4C
MAML1	KPNB1	ZC3H7A	PQLC3
NAB2	MAGEE1	AK5	PTK2B
PSEN1	PIK3CG	AMER1	PTK7
TACC1	PLAU	BAG4	RASGEF1A
TCTA	RANBP2	BCAS4	RHBDF2
WRN	RPN2	BCL10	RRM2B
ZNF24	RUNX1	BCL11B	43713-SEPT5
DPM1	SGK1	BCL2A1	SIN3A
KTN1	ATF1	BRD1	SMARCA1
AUTS2	ATRNL1	BRIP1	SOX2
CEBPA	CDK9	C11orf30	SPOP
KRAS	CDKN1A	CCNE1	SPTAN1
RABEP1	CENPF	CDC25A	TEAD2
RHOA	CHCHD7	COG5	TERF2
XPA	CSNK1G2	CREBBP	TOP2A
CSF1	CTSA	CTLA4	TPM4
NDC80	DDX6	CXXC4	TTK

RPS21	ELF4	FAM19A5	USP42
SOD2	FEN1	FANCI	WDR70
TNFRSF14	FLNA	GAS7	ABCC3
ACVR1B	FNBP1	GMPS	ACVR1C
FOXP1	FOS	GNAI1	AKT1
PSIP1	FUT1	GPR124	ALDH1A1
XPC	GNA11	GSN	ARHGEF7
ZMYND11	H2AFX	HIST1H2AM	ATRX
ZNF331	HIST1H2AL	INHBA	CCND2
ARID2	HIST1H3B	ITGB3 (CD61)	CDH1
BCL9	ICAM1	KDM6A	CDK8
CAPZB	IL7R	KIT (CD117)	CDKN2B
CCND3	JAK1	LCP1	CEBPD
DEK	KAT6B	LMBRD1	CHD6
EP400	LRPPRC	LMO1	CRTC3
HIST1H2BK	MAP3K1	LOX	CSF3R
HSPA4	MLLT11	LRP5	DGKI
IKZF1	NCKIPSD	MADD	DIS3L2
LRRC37B	NFKB1	MALT1	DNM3
MYO1F	NT5C2	MAP2K1	EGF
NF2	PCSK7	MAP2K6	EGR2
NUP214	PPP3CC	MUTYH	ENPP2
PALB2	PRKCD	NFIB	EPHB6
PBRM1	RASGRP1	NSD1	ERCC1
PICALM	RELN	PAFAH1B2	ERCC3
SMAD4	RPS6KA3	PI4KA	FANCF
TFRC (CD71)	SNW1	PTPN6	FGFR1
VEGFC	STAT4	RB1	FHIT
ZNF217	TAF1	SBDS	FRK
ATG13	TERT	SLX4	HES5
AURKA	TMEM127	SMAP1	HIST1H2BO
BTG1	TPD52L2	SMC3	HSP90AA1
CARD11	TPR	SPEN	ID1
CASP7	TRIM24	SRSF2	IDH2
CASP8	WHSC1L1	TFEB	IKZF3
CDC42	ACSL6	TOP2B	IL3
CDK1	ANKRD28	USP7	INPP4A
CNOT2	CCNA2	WIF1	ITGAV
EGR1	CEP85L	ZMYM2	KDM2B
ELL	CXCL8	ZRSR2	KLK7
EPC1	DDIT3	ACKR3	MATK
EPCAM	DDX20	AKAP6	MET
EPOR	DLEC1	AKT3	MITF
ERCC6	ELK4	ALDOC	MKL1
EZR	FHL2	ARHGAP26	MLH1
FBXO11	GLI3	ASPH	MSH6

FSTL3	HEY1	ATR	NACA
HDAC5	MAP3K7	BCL6	NCOR2
HIF1A	MAPK8	BRD4	NFKBIA
HIST1H2BC	PATZ1	BRWD3	NUMA1
IRS2	PAX8	CD79A	PAK6
MALAT1	PIK3R1	CDC14B	PBX1
MKI67	PMS2	CDX2	PDCD11
P2RY8	RTEL1-TNFRSF6B	CIRH1A	PEG3
PPAP2B	TNF	CIT	PFDN5
STAT1	VEGFA	COL11A1	PPP2R2B
THRAP3	XRCC6	CTNNA1	PTPN11
TRIM33	ZNF687	CYP1B1	PTPRA

Shown are the 400 genes identified in pre-transplant marrow that associated with the development of aGvHD. Genes are listed in order of expression.

Table S6: *700 Genes Predicting Overall Survival in Pre-transplant Samples*

1-100	101-200	201-300	301-400	401-500	501-600	601-700
ANKRD28	TRIM24	DIRAS3	CREBBP	IFRD1	JARID2	DKK1
PDK1	CHST11	DNM3	SUZ12	PATZ1	PRKACA	ELN
PIK3CB	GATA1	PAX8	CCNG1	CD22	RMI2	FZD8
ARID2	PKM	PPAP2B	LMO2	FLT3LG	DTX1	RGS7
APC	SH3GL1	STIL	CCNB1IP1	HSPA2	FLNA	RUNX2
MAPK3	SP3	TFRC (CD71)	INPP5A	ITPKA	FANCE	TRHDE
NSD1	PTBP1	POT1	WNT11	WT1	FLT3	BCL2A1
PPP1CB	ANGPT1	RAD21	ZNF521	CBLB	HOXA10	GPHN
TFG	C3orf27	CDC73	JAZF1	SARNP	LAMA5	IKBKE
PPP4C	DNM1	DTX4	RRM1	SMC1A	LRRC7	POLD1
SEC31A	LHX4	IL21R	ZNF207	HIST1H2BC	PPARGC1A	POLR2H
ABL1	TAF1	MLLT3	ANLN	EPHB1	PROM1	REEP3
SF3B1	TPM4	CEBPA	C11orf95	HIST1H3B	PLAT	USP5
FLI1	TAOK1	KANK1	WIF1	NCKIPSD	TNC	XPC
LRRC37B	SRSF2	CACNA1F	CTLA4	EPHA2	AURKA	PTK2B
SPEN	ETV5	ASPSCR1	DDB2	FAM19A5	CD74	TAL1
DDX20	MNX1	CARM1	DEK	HOXA9	CHUK	U2AF2
SLC7A5	MSN	PDCD1 (PD-1)	INPP4A	MAP2	FAM46C	XPO1
KDM6A	MALT1	UBE2B	TFPT	STL	FBXW7	INPP5D
RANBP17	GIT2	CDK9	WHSC1	AXIN1	LRP5	KIAA0232
YWHAE	LASP1	PSMD2	HIST1H2BO	CD8A	MAP2K7	MAPK1
PCBP1	CDKL5	CDKN1C	MAML1	FASLG	SDHD	NOTCH1
CRTC1	FHIT	NPM1	OFD1	MLLT4	BCL10	AHI1
BRCA2	GATA2	IL1RAP	CALR	PPP2R2B	HIST1H2AG	EGR1
IGFBP2	MMP9	PPP2R4	C10orf55	TAF15	JAK3	GPR34
TET2	DDIT3	WASF2	MB21D2	WHSC1L1	MYC	GRID1
TEC	EP400	LPP	MPL	CDK8	NIPBL	KLK2
CDK7	YPEL5	IGF1R	PTCRA	CSF3R	PIK3CG	SFRP4
LYN	KRAS	ASXL1	VEGFC	PIK3CD	CAMK2G	SH3GL2
MAP2K2	TCF7L2	NEDD4	ACKR3	HIPK2	MAP2K4	SLCO1B3
AFF4	KIF5B	43713-SEPT5	FGFR3	CTNNB1	SBDS	TEAD2

CCT6B	BDNF	WDFY3	AIP	VHL	SMC3	CHMP2B
HIST1H2AC	EZR	MITF	BAX	DCLK2	STAG2	DICER1
ARID1A	MIB1	MYCN	MCL1	FGF9	ABCC3	MELK
TMEM127	TCEA1	PHF6	MYO18A	IL2	OLR1	MNAT1
NT5C2	STAT5A	SFRP2	USP7	PDGFA	CTRB2	NFKBIA
SETD2	ZMYM2	CDKN1A	ID1	SRC	AXL	PIM1
BAG4	CTSA	CNOT2	RPA3	DDR2	CCDC88C	RNF213
CKB	HOXA3	MAP2K1	TNFRSF10D	FRK	CDC25A	43714-SEPT6
PC	LINC00598	NDRG1	ERG	PLA2G5	EDNRB	MLLT1
SIN3A	CCND2	TERF2	NCOR2	RTEL1	EWSR1	NCOA2
LAMP2 (107B)	GID4	IRF2BP2	SPTBN1	SMARCA1	IRF4	PTEN
ARRDC4	ATR	FSTL3	TCF12	AKT2	LRRC59	CD58
HHEX	BCL6	KIAA1549	WDR1	BCL11B	RLTPR	MAD2L1
CBL	USP42	MDM2	BCAS4	GATA3	ACVR1B	TMEM230
SP1	CNBP	FGF10	CIRH1A	GNA11	APOD	IRS2
KDM1A	DLL1	HOXA11	GSTT1	MADD	ASMTL	RICTOR
PPP2R1A	TOP1	CDH1	CDKN2B	MKL1	CCDC6	HSPA5
BCL9	EP300	DGKZ	EPO	NONO	HIST1H2BJ	NPM2
STX5	CTNNA1	KAT2B	FGF2	TGFBR3	TFE3	AGR3
USP6	MSH3	NF2	NFIB	MBTD1	BCL3	CMKLR1
PPP3CB	RPL22	PML	PAK6	GRB2	BCOR	FANCC
CRKL	FCGR2B	CHIC2	ETS2	PAX5	CKS1B	HEY1
LMBRD1	HNRNPA2B1	FAF1	IL1B	PDGFB	SMARCA5	NCOA4
MTCP1	NEURL1	HDAC1	LGALS3	BCL2L1	HIPK1	PDCD11
RPN1	TRPS1	TMEM30A	MYBL1	ETS1	ESR1	PLCG1
SUFU	CASP3	CAPRIN1	SIRT1	RAP1GDS1	HIST1H4I	THBS1
SYK	ERC1	NBEAP1	WDR70	TNFRSF10B	KLF4	TRAF5
CUX1	FANCD2	YTHDF2	KLHL6	TRAF2	AKAP12	FZD7
PTK2	MDC1	EGF	THADA	PLCG2	IL13RA2	GLI1
RCOR1	MED12	CD70	TYK2	PTPN11	KALRN	LINGO2
HGF	SPRY2	CCNA2	ABL2	PHF23	RELN	LRP1B
ACSL6	DACH1	FEN1	GNAQ	RPN2	TBX15	NGFR
ICK	SMAD6	NRAS	SMARCB1	TTK	ZFPM2	PCNA-AS1

XIAP	SNHG5	ELF4	BRD1	ACVR1C	BAIAP2L1	SOCS2
PAK1	IL13	FNBP1	SMAD2	BCL11A	NGF	ATP1B4
CLTCL1	OLIG2	PI4KA	ERLIN2	ITGB3 (CD61)	SOX11	BRSK1
NCAM1 (CD56)	TBL1XR1	PRKCD	GAS7	LEFTY2	DUSP22	FCGBP
PIK3R2	TCF3	CD79B	NR4A3	MIPOL1	EZH2	FLT1
TTL	BRD3	ETV6	KAT6B	STAT1	GNAS	SRRM3
DUSP2	GSK3B	GFAP	NFE2L2	ATP6V1G2-DDX39B	NAPA	ERCC5
SPTAN1	PRKCB	SRGAP3	WEE1	HOXA13	SNAPC3	HIST1H1D
NR3C1	XPA	ARHGEF12	CNTRL	LINC00982	SPECC1	MAFB
ALDH2	CCDC28A	BCL2	ATL1	IL12RB2	TLR4	RHBDF2
XBP1	FUS	CSF1	RASGEF1A	RAC3	CDKN1B	RPTOR
PLAG1	SOS1	FGFR1OP2	ADM	SPP1	PICALM	NUTM2B
KSR1	LTBP1	FOXO3	DNMT1	SSX2B	BTG2	SOD2
PBRM1	METTL7B	FOXO4	GATA6	HIST1H2BK	CAMTA1	IDH1
CREB3L2	FLCN	HMGB1	LYL1	SNX29	GRB10	GMPS
MLLT10	RECQL4	MATK	MYD88	STYK1	GSN	ALDOC
DGKI	TNFRSF11A	NBR1	SETBP1	TRIM27	HDAC2	GTSE1
C2CD2L	ZRSR2	NDUFAF1	ARAF	KMT2D	SDHA	IRF1
FAS	MAX	TERF1	DDX39B	SRF	FGF8	MBNL1
CDK12	ZNF687	TPM3	HDAC7	TRIM33	STAT4	PAFAH1B2
POM121	COX6C	ZBTB16	KMT2B	FANCM	ATRNL1	SNX9
CYP1B1	MEF2D	FBXO11	MAP3K1	MEAF6	DKK2	AR
STK11	CARD11	RPS6KA3	NUMA1	XRCC6	PRDM16	DLEC1
TFDP1	CCND1	FH	BRIP1	FGFR1OP	EGR2	FOS
MAF	IKBKB	MAP2K6	MRE11A	FRYL	FAM64A	MDS2
MAPK9	MAP2K3	RAD51B	BCORL1	LRPPRC	HLF	SFPQ
STAT3	BIRC6	TGFBR2	ERBB2	AHR	VCAM1	BIVM-ERCC5
SDHAF2	FANCB	BCL7A	NFKB1	FGF13	AKT1	GRIN2A
C11orf1	RHOA	FAM19A2	VTI1A	PLCB4	2-Sep	ID4
MEF2BNB-MEF2B	SH3BP1	S1PR2	ACACA	TGFB2	ZNF331	METTL18
NR6A1	RREB1	TIRAP	EPS15	AK2	BAZ2A	PRDM7
PCM1	FOXP1	DAB2IP	ATG5	BCL2L2	GANAB	RERG
SYP	NUP214	PTPRR	RELA	PRF1	ERCC1	TLX1

ACSL3	RTN3	COG5	WSB1	SDC4	KMT2C	XKR3
CDC42	ATF3	BCR	AFF3	CLP1	RBM15	FGFR1
SORT1	COL9A3	PFDN5	FRS2	HIST1H1E	RHOH	IL15

Shown are the 700 genes identified in pre-transplant marrows that associated with overall survival. Genes are listed in order of expression.
